# Production of indole by *Corynebacterium glutamicum* microbial cell factories for flavor and fragrance applications

**DOI:** 10.1186/s12934-022-01771-y

**Published:** 2022-03-24

**Authors:** Melanie Mindt, Arman Beyraghdar Kashkooli, Maria Suarez-Diez, Lenny Ferrer, Tatjana Jilg, Dirk Bosch, Vitor Martins dos Santos, Volker F. Wendisch, Katarina Cankar

**Affiliations:** 1 Business Unit Bioscience, Wageningen Plant Research, Wageningen University & Research, Wageningen, The Netherlands; 2 Axxence Aromatic GmbH, Emmerich am Rhein, Germany; 3 Laboratory of Systems and Synthetic Biology, Wageningen University & Research, Wageningen, The Netherlands; 4 Genetics of Prokaryotes, Faculty of Biology & CeBiTec, Bielefeld University, Bielefeld, Germany; 5 Laboratory of Bioprocess Engineering, Wageningen University & Research, Wageningen, The Netherlands

**Keywords:** Indole, Flavor and fragrance applications, Bioprospecting, Tryptophanase, *Corynebacterium glutamicum*, In situ product recovery, Bioconversion, Tryptophan

## Abstract

**Background:**

The nitrogen containing aromatic compound indole is known for its floral odor typical of jasmine blossoms. Due to its characteristic scent, it is frequently used in dairy products, tea drinks and fine fragrances. The demand for natural indole by the flavor and fragrance industry is high, yet, its abundance in essential oils isolated from plants such as jasmine and narcissus is low. Thus, there is a strong demand for a sustainable method to produce food-grade indole.

**Results:**

Here, we established the biotechnological production of indole upon l-tryptophan supplementation in the bacterial host *Corynebacterium glutamicum*. Heterologous expression of the tryptophanase gene from *E. coli* enabled the conversion of supplemented l-tryptophan to indole. Engineering of the substrate import by co-expression of the native aromatic amino acid permease gene *aroP* increased whole-cell biotransformation of l-tryptophan to indole by two-fold. Indole production to 0.2 g L^−1^ was achieved upon feeding of 1 g L^−1^ l-tryptophan in a bioreactor cultivation, while neither accumulation of side-products nor loss of indole were observed. To establish an efficient and robust production process, new tryptophanases were recruited by mining of bacterial sequence databases. This search retrieved more than 400 candidates and, upon screening of tryptophanase activity, nine new enzymes were identified as most promising. The highest production of indole in vivo in *C. glutamicum* was achieved based on the tryptophanase from *Providencia rettgeri.* Evaluation of several biological aspects identified the product toxicity as major bottleneck of this conversion. *In situ* product recovery was applied to sequester indole in a food-grade organic phase during the fermentation to avoid inhibition due to product accumulation. This process enabled complete conversion of l-tryptophan and an indole product titer of 5.7 g L^−1^ was reached. Indole partitioned to the organic phase which contained 28 g L^−1^ indole while no other products were observed indicating high indole purity.

**Conclusions:**

The bioconversion production process established in this study provides an attractive route for sustainable indole production from tryptophan in *C. glutamicum*. Industrially relevant indole titers were achieved within 24 h and indole was concentrated in the organic layer as a pure product after the fermentation.

**Supplementary Information:**

The online version contains supplementary material available at 10.1186/s12934-022-01771-y.

## Background

Indole is a nitrogen containing heterocyclic aromatic compound, first found in an indigo reduction process. It is widely distributed in the natural environment, where it was identified in animal feces, coal tar, and essential oils of several flowers [1–4]. The human nose recognizes indole and many of its derivatives at low odor detection threshold [5]. At low concentrations, indole is often described as having a sweet and floral odor typical for jasmine flowers. It also contributes to the flavor of food and the aroma of perfumes and is therefore used as flavor enhancer and fragrance in the food and cosmetics industries [6]. Bulk production of indole is mainly based on isolation from coal tar. Also its chemical synthesis was subject of a plethora of studies of organic chemists [for reviews see 7, 8]. The major drawback of these indole production methods for the flavor and fragrance industries is its classification as synthetic product.

In nature, indole acts as signaling molecule with significant roles within prokaryotic and eukaryotic communities [9]. Some plants, such as maize and rice, liberate indole as priming signal to activate defense systems in response to herbivore attacks [10, 11]. Indole secreted by soil bacteria was shown to promote growth of plants like *Arabidopsis thaliana* and Chinese cabbage [12, 13]. In bacteria, indole influences multiple aspects of their physiology, such as biofilm formation and virulence [14]. Exposure to indole induced expression of multiple transport system genes, and consequently increased drug resistance of *Escherichia coli* [15] and *Salmonella* species [16]. Transport systems for indole in the natural indole producing bacterium *E. coli* were described, however, indole can also rapidly cross the cytoplasmic membrane by diffusion [17].

**Fig. 1:**
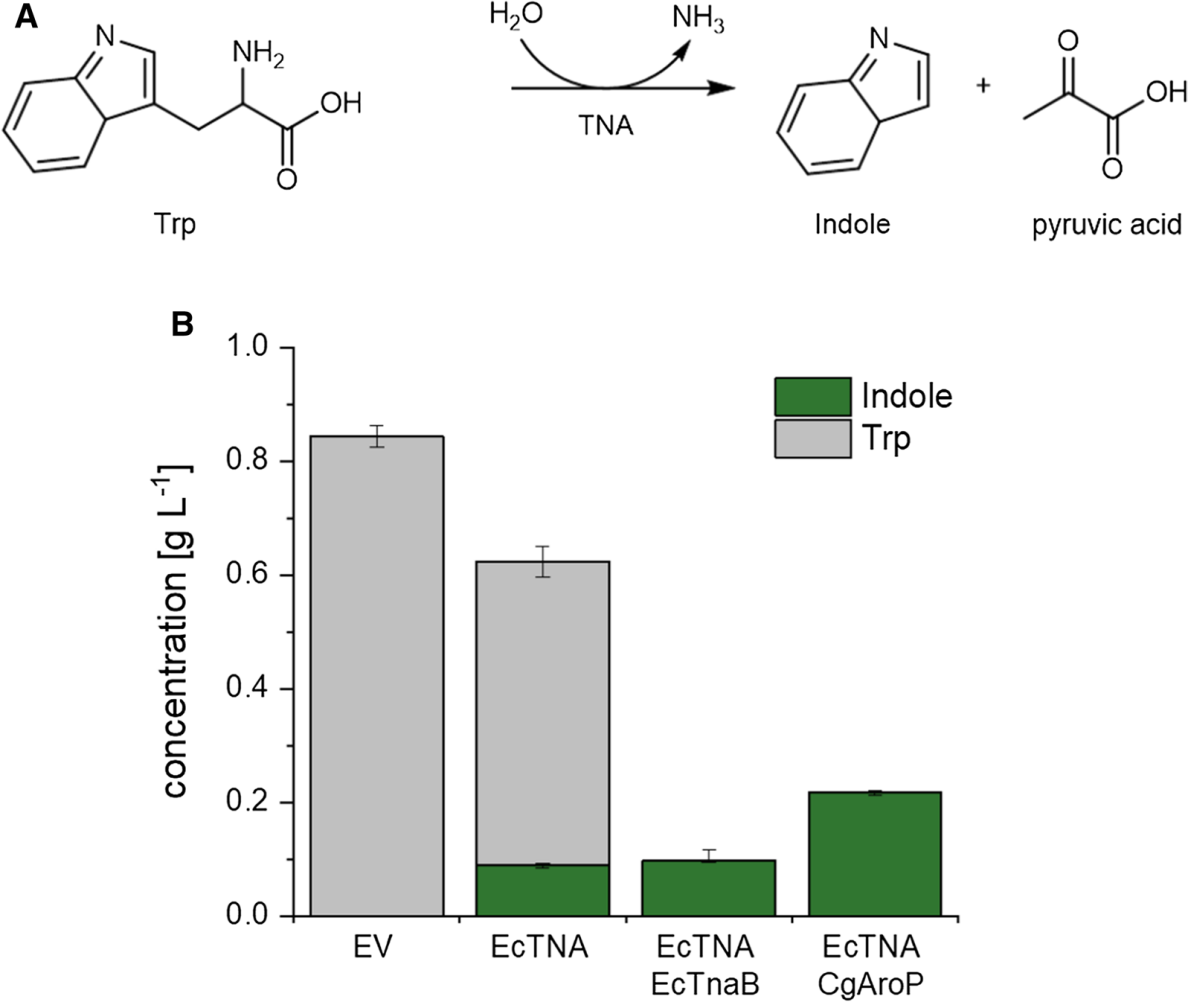
Proof of principle for biotechnological indole production in C. glutamicum C1*. Schematic view of TNA reaction for indole production (**A**). Production of indole upon expression of *E. coli tnaA* and Trp importer genes *tnaB* and *aroP* (**B**). Strains were grown in minimal medium CGXII supplemented with 40 g L^−1^ glucose and 1 g L^−1^ Trp . EV: empty vector pGold; EcTNA: TNA derived from *E.* *coli*; EcTnaB: Trp permease from* E. coli*; CgAroP: aromatic amino acid permease from *C. glutamicum*

Currently, different biosynthesis pathways for indole have been described. Synthesis of indole as an intermediate of l-tryptophan (Trp) biosynthesis is the most prevalent metabolic pathway [18]. Tryptophan synthase catalyzes a two-step conversion of indole-3-glycerolphosphate to Trp with indole as intermediate, however, due to tight allosteric regulation of its subunits, indole release is prevented [19, 20]. Indole is not only part of the primary metabolism in all domains of life, it also participates in the secondary metabolism of several microorganisms and plants. In the secondary metabolism of plants, indole production from indole-3-glycerolphosphate is catalyzed by indole-3-glycerol-phosphate lyases, which do not operate in a heteromeric form which allows immediate release of indole [21]. In bacteria, tryptophanase (TNA) hydrolyzes Trp in a β-elimination reaction yielding indole, pyruvate and ammonia (Fig. 1A). The carbon-carbon bond of Trp is cleaved such that indole is released and an unstable enamine/imine intermediate forms, which spontaneously decomposes to pyruvate and ammonia via hydrolytic deamination [22]. TNAs operate as homotetramers, in which each subunit binds one molecule of pyridoxal-5-phosphate (PLP) and a monovalent cation, under physiological conditions K^+^, to enable TNA activity [23, 24]. Indole production upon tryptophan addition was subject to a study designed to evaluate the relevance of externally added Trp to the natural indole producer *E. coli* [25]. In this study titers up to almost 6 mM (corresponding to 0.7 g L^−1^) were described, an amount which is in range of the concentrations so far examined for their physiological effects [25].

Rapid advances in synthetic and systems biology provided a broad repertoire of editing tools to create cell factories to produce bulk and fine chemicals. One of the major production hosts especially for amino acids and derived compounds is the natural glutamate producer *Corynebacterium glutamicum* [26]. Next to several other targets, *C. glutamicum* strains were re-designed for production of aromatic compounds such as shikimate [27], Trp [28], and also the anthranilate derivatives *O*-methylanthranilate [29] and *N*-methylanthranilate [30]. Although *C. glutamicum* does not naturally produce indole, its physiological response to indole exposure has been investigated recently [31]. This study focused on tolerance of the wild-type strain *C. glutamicum* ATCC 13032 and the derived genome-reduced chassis strain C1* [32]. Both *Corynebacterium* strains showed perturbed growth when exposed to indole. Interestingly, the effect of indole addition to the chassis strain C1* was minor compared to the wild-type strain. Transcriptome analysis of the wild-type strain cultivated in presence of indole revealed changed expression levels of genes involved in metabolism of aromatic compounds such as the *p*-cresol and cytochrome *bd* catabolism, but also genes related to iron and copper. It is notable that several genes affected by the presence of indole in the wild-type strain are deleted in the chassis strain C1* [31, 32].

The objective of this work was to establish a biotechnological production process for food-grade indole in *C. glutamicum* C1*. The chassis strain is favored over its wild-type ancestor due to its higher tolerance towards indole and the lack of a flavin-dependent monooxygenase known to oxidize indole to indoxyl, which spontaneously dimerizes to the pigments indigo and indirubin [33]. Introduction of the well-studied TNA from *E. coli* into *C. glutamicum* led to successful formation of indole. Prospecting of bacterial genomes for TNA genes (*tnaA*) and subsequent screening retrieved several new candidates with TNA activity that were used for indole production. Process optimization strategies needed to be applied to establish indole production in a gram per liter scale.

## Results and discussion

### Proof-of-principle for biotechnological indole production by *C. glutamicum*

Since *C. glutamicum* ATCC 13032 has not been reported to produce indole and lacks a TNA gene, heterologous expression of a TNA gene is required to establish Trp hydrolysis to indole. The well-studied TNA from *E. coli* (*EctnaA*) was cloned into the expression vector pGold and used to transform the chassis strain C1*. Supernatants of C1* carrying either the empty vector pGold or pGold-*EctnaA* cultivated in minimal production medium CGXII did not lead to indole accumulation. However, no Trp production was observed either, which indicates a low de novo production of the substrate and, as consequence, no product formation. Addition of 1 g L^−1^ Trp to cultures expressing *EctnaA* led to accumulation of 0.1 ± 0.01 g L^−1^ indole, yet, more than 60% of Trp remained in culture supernatants (Fig. 1B).

In *E. coli*, Trp uptake was found to be essential for conversion of externally added Trp to indole, which was enabled by expression of the endogenous Trp permease gene *tnaB* [25, 34]. To facilitate the substrate uptake in C1*, the Trp permease gene *tnaB* from *E. coli* or the endogenous aromatic amino acid permease gene *aroP* were co-expressed with *EctnaA*. Plasmid-based expression of both transporter genes improved the uptake of the substrate (Fig. 1). Co-expression of *tnaB* with *EctnaA* resulted in decreased Trp concentrations in the medium, however, did only have minor effects on substrate conversion. On the contrary, the plasmid-based expression of the endogenous permease gene *aroP* showed a 38 mol-% conversion of the substrate to a final titer of 0.22 g L^−1^ indole within 24 h, while Trp was almost depleted in culture supernatants. Analysis of biomass and culture supernatants separately showed presence of indole almost exclusively in culture supernatants, showing efficient export out of the *C. glutamicum* cell.

**Fig. 2:**
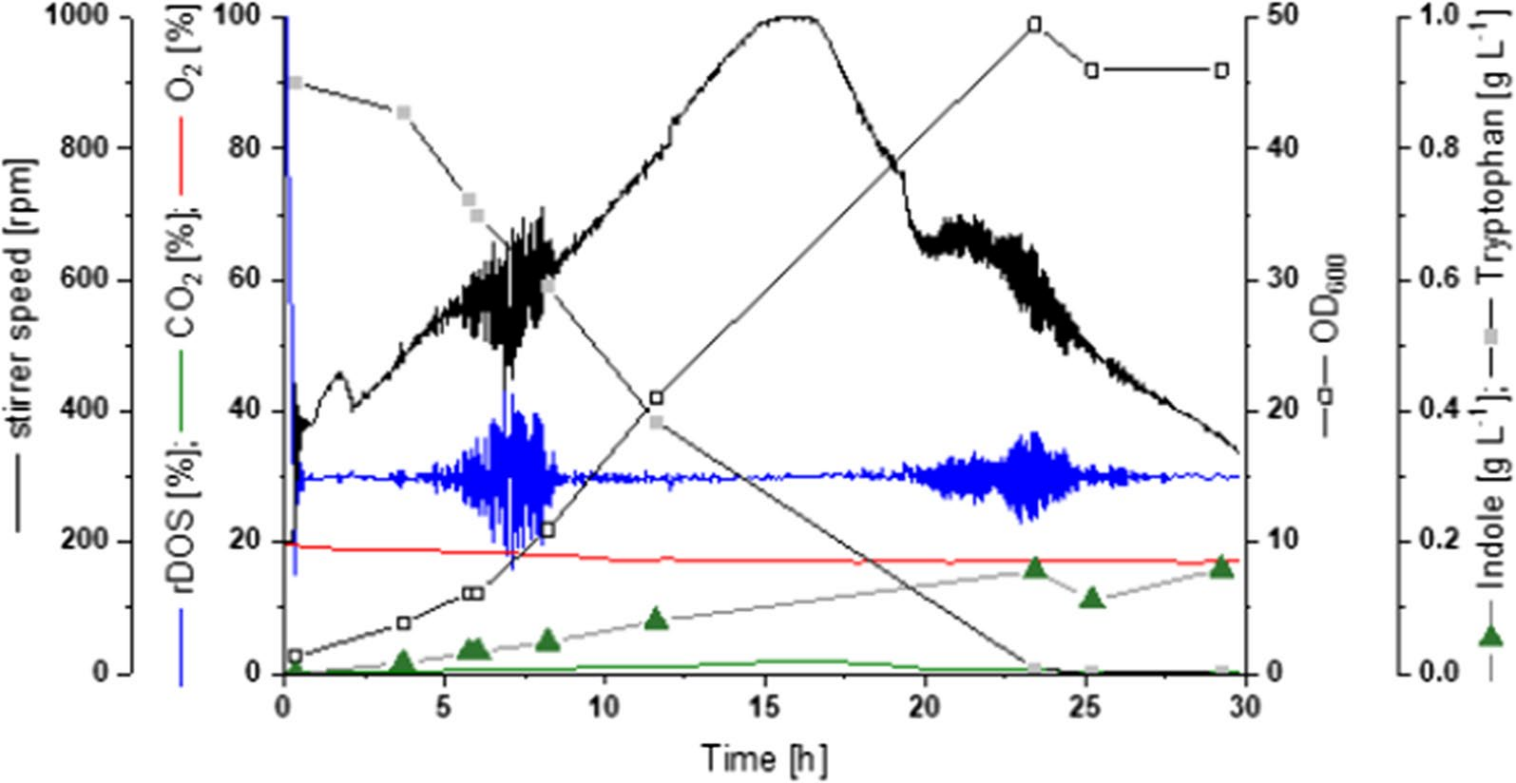
Bioreactor cultivation of Trp biotransformation is depicted as one representative of three comparable cultivations

For successful biotechnological production of indole, not only the microbial host, but also the culture conditions must prove suitable. For this purpose, the biotransformation process was transferred from shake flask to laboratory scale bioreactor cultivation in 1 L vessels. The bioconversion strain C1*(pGold-*EctnaA*-*CgaroP*) was cultivated in modified CGXII medium supplemented with 40 g L^−1^ glucose and 1 g L^−1^ Trp in batch mode. The added Trp was depleted almost completely 24 h after inoculation and at the same time the highest indole titer (0.21 ± 0.01 g L^−1^) was reached (Fig. 2).

Analysis of culture supernatants proved no substantial byproduct formation during the process (Additional file 1: Fig. S1). Next, the off-gas of the bioreactors was investigated for the presence of indole to evaluate whether the intense aeration has led to indole losses to the gas stream. Aeration of the cultures in the bioreactor is achieved by applying a stream of air added to the bottom of the vessel in conjunction with vigorous agitation. Therefore, the off-gas was analyzed by first capturing the air stream for 15 min with a tube equipped with a hydrophobic matrix (Tenax) and subsequent thermal desorption gas chromatography coupled to a mass spectrometer. Simultaneous to highest indole accumulation in the medium the highest product loss was detected 24 h after inoculation, yet, the off-gas contained only 5 ± 3 µg h^−1^ of indole, which is negligible compared to the indole titer produced. Thus, no major product loss to the gas phase is to be expected in production processes. As a proof-of-principle, production of indole by *C. glutamicum* biotransformation was shown in lab-scale bioreactor cultivation. Both the host and the process proved to be suitable for biotechnological indole production.

### Bioprospecting to identify new TNAs

To test if nature provides TNAs more suitable than the benchmark EcTNA, publicly available bacterial genomes were screened for presence of TNA enzymes. Creating a non-redundant list of TNA sequences led to the identification of 407 protein sequences annotated with the EC number 4.1.99.1. All candidate sequences contained the β-eliminating lyase PFAM domain PF01212, typical for TNA enzymes. In addition, with a few exceptions, the genes code for enzymes with a theoretical mass of ~ 50 kDa. The average length of the considered sequences was 471 ± 34 amino acids and ranged from 209 to 568. Exploration of the sequence identity between the considered sequences showed that they could be grouped in 14 clusters, as shown in Additional file 1: Fig. S2. Cluster one contains a single sequence, originating from *Corynebacterium marinun*, with the shortest length of all considered sequences (209 amino acids), therefore, it was discarded from further analysis. The second cluster contains the sequence of the *EctnaA.* Candidate sequences from clusters 2 to 14 were selected by considering characteristics of the host species such as lack of reported pathogenic status or literature evidence (if available) on the indole production potential of the organism and to achieve broad sequence diversity (Additional file 1: Fig. S2). By these criteria, 14 new TNA candidates from 13 different clusters were chosen for testing in strain C1* (for gene sequences see Additional file 2). Candidates belonging to several taxonomic clades were chosen. This includes candidates from *Corynebacterium xerosis*, *Escherichia hermannii*, the photosynthetic bacterium *Rhodobacter sphaeroides*, the enterobacterium *Proteus vulgaris*, the salt-tolerant *Providencia rettgeri*, *Mageeibacillus indolicus*, and *Clostridiales bacterium*, the latter two bacteria are members of the class of clostridia. Also the characterized *tnaA* from *Proteus vulgaris* was included in the study [35].

### In vivo and in vitro characterization of new TNA candidates

**Fig. 3:**
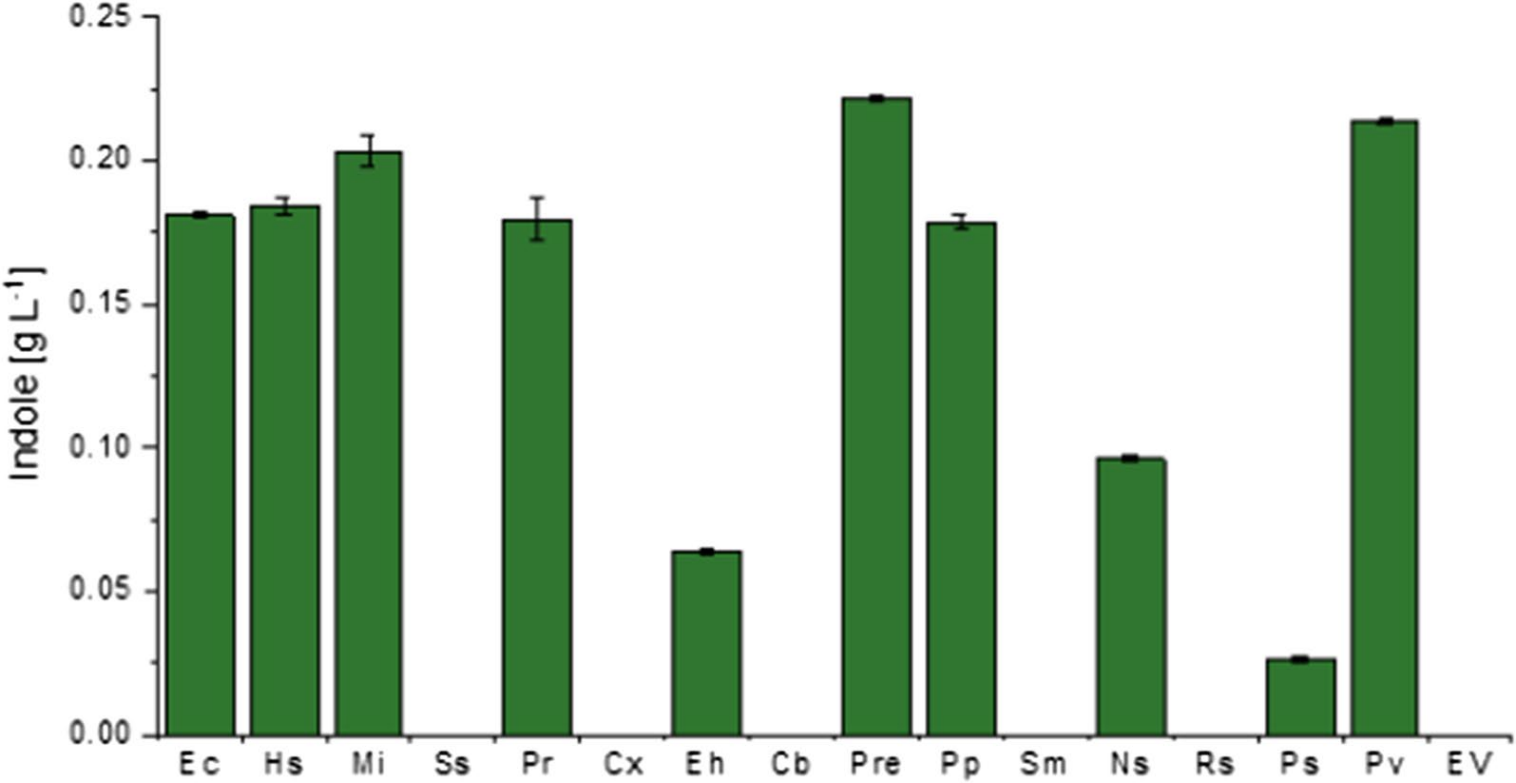
Indole production by strains equipped with different TNA enzymes in C1* upon 1 g L^−1^ Trp supplementation. Samples were taken from duplicate cultures 40 h after inoculation. Means and arithmetic errors of duplicate cultures are shown. Ec: *tnaA* from *E. coli*; Hs: *tnaA* from *Histophilus somni*; Mi: *tnaA* from *Mageeibacillus indolicus*; Ss: *tnaA* from *Synechocystis* sp.; Pr: *tnaA* from *Proteiniclasticum ruminis*; Cx: *tnaA* from *Corynebacterium* xerosis; Eh: *tnaA* from *Escherichia hermannii*; Cb: *tnaA* from *Clostridiales bacterium*; Pre: *tnaA* from *Providencia rettgeri*; Pp: *tnaA* from *Prevotella pallens*; Sm: *tnaA* from *Sciscionella marina*; Ns: *tnaA* from *Nocardioides* sp.; Rs: *tnaA* from *Rhodobacter sphaeroides*; Pp: *tnaA* from *Photobacterium* sp.; Pv: *tnaA* from *Proteus vulgaris*; EV: empty vector pGold

The respective 14 TNA genes were ordered as synthetic genes after codon optimization for optimal expression in *C. glutamicum*. The genes were cloned into the expression vector pGold and expressed in the chassis strain C1*. All strains were cultivated in minimal medium CGXII supplemented with 40 g L^−1^ glucose and 1 g L^−1^ Trp. Expression of nine new *tnaA* candidates supported indole production. Importantly, six strains, i.e. those carrying *tnaA* from *Histophilus somni, M. indolicus, Proteiniclasticum ruminis*, *P. rettgeri*, *Prevotella pallens*, and *P. vulgaris* produced indole to similar or higher concentrations than the well-characterized EcTNA (Fig. 3). Four of these candidates (HsTNA, MiTNA, PvTNA and EcTNA) were also visible in the soluble protein fraction of the respective C1* strain, yet, PrTNA, PreTNA and PpTNA were not (Additional file 1: Fig. S3). On the contrary, *tnaA* from *C. bacterium* was expressed, but did not convert Trp to indole, thus not encoding an active TNA (Fig. 3).

**Fig. 4:**
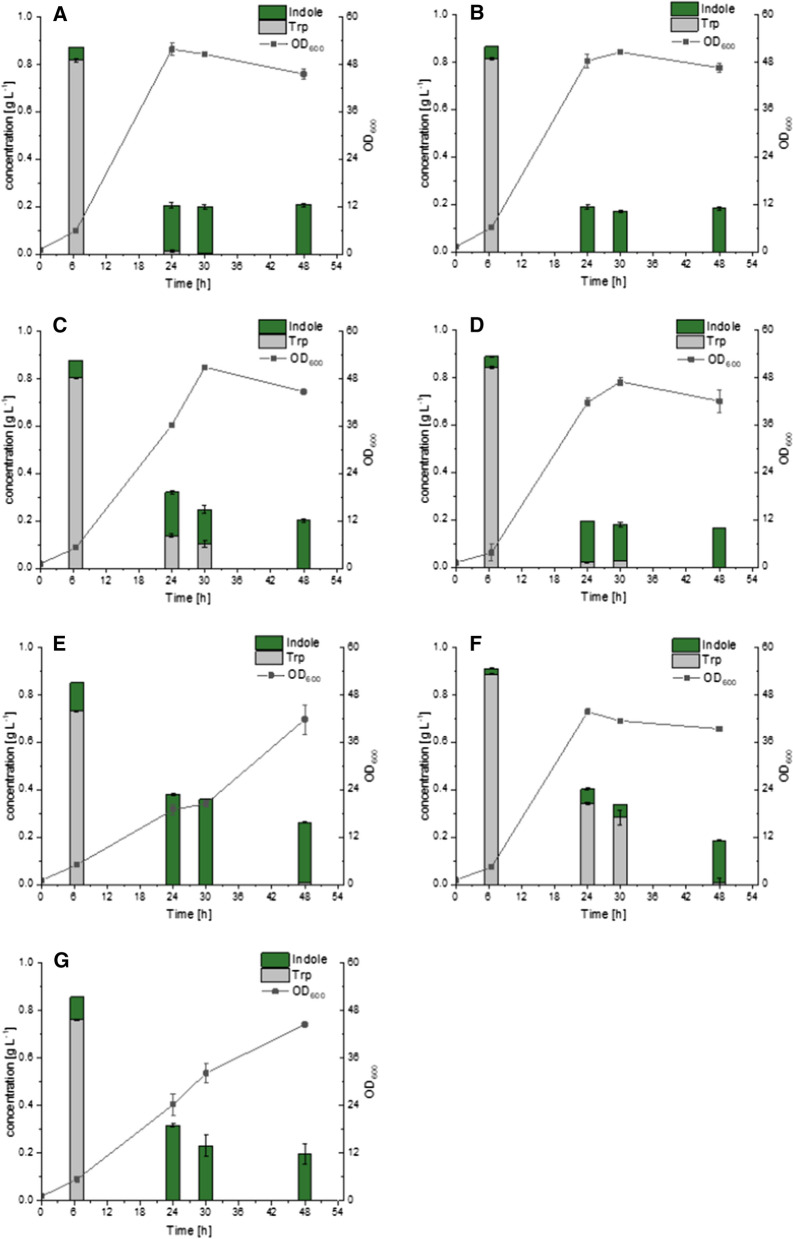
Biotransformation of Trp to indole using strains equipped with seven different TNA enzymes. The six most promising TNA candidates were co-expressed in C1* with *CgaroP* and their performance was compared to *tnaA* from *E. coli.* **A** *tnaA* from *E. coli*; **B** *tnaA* from *Histophilus somni*; **C** *tnaA* from *Mageeibacillus indolicus*; **D** *tnaA* from *Proteiniclasticum ruminis*; **E** *tnaA* from *Providencia rettgeri*; **F** *tnaA* from *Prevotella pallens*; **G** *tnaA* from *Proteus vulgaris*. Means and arithmetic errors of duplicate cultures are shown

Next, the six best *tnaAs* were co-expressed with the endogenous aromatic amino acid permease gene *aroP* to overcome Trp import limitation. Strains were cultivated in CGXII minimal medium supplemented with 1 g L^−1^ Trp and complete Trp depletion was observed 48 h after inoculation. Indole production was observed for all six tested TNAs (Fig. 4).

Strains expressing *PrtnaA* and *PptnaA* showed less indole accumulation at 0.16 and 0.18 g L^−1^ in 48 h, respectively, while analysis of culture supernatants of the strain expressing *PretnaA* proved highest indole production with a final titer of 0.25 g L^−1^. The maximal indole concentration of most strains was reached within 24 h after inoculation. The benchmark strain expressing *EctnaA* showed its indole concentration peak (0.19 g L^−1^; similar to mol-% conversion of 34% of Trp) 24 h after inoculation. Interestingly, the two strains expressing *PvtnaA* and *PretnaA* reached even higher indole production (0.32 and 0.38 g L^−1^, respectively) with a mol-% conversion of Trp of more than 50% (55 mol-% and 66 mol-%, respectively). The conversion of Trp to indole could be noticed already 6.5 h after inoculation. While C1*(pGold-*EctnaA-CgaroP*) secreted 0.06 g L^−1^ indole within the first 6.5 h, the two strains expressing *PvtnaA* or *PretnaA* secreted almost twice as much indole in the same period. However, indole concentrations in supernatants of the strain expressing *EctnaA* remained stable after reaching the maximum, whereas indole concentrations in supernatants of strains expressing either *PvtnaA* or *PretnaA* decreased by almost 50% in the later stage of the process.

Kinetic constants for four purified TNAs (EcTNA, MiTNA, PvTNA and PreTNA) were evaluated (Table 1). Protein production in *E. coli* BL21(DE3) was performed by heterologous expression of the respective gene encoded on the expression vector pACYCDuet-1. Addition of an N-terminal 6x His-tag enabled subsequent protein purification by affinity chromatography. By coupling the TnaA reaction to NAD-dependent lactate dehydrogenase, which reduces the released pyruvate to lactate, enzymatic kinetics were recorded spectrophotometrically via NADH. PvTNA showed the highest substrate affinity for Trp, while the highest turnover number 2.41 ± 0.06 s^−1^ was achieved by MiTNA. In vitro catalytic efficiency of PvTNA was the highest with 26 ± 6 mM^−1^ s^−1^, the second best catalytic efficiency was observed for EcTNA (Table 1). It is noteworthy that PreTNA showed low affinity for Trp and also low turnover rates in vitro, however, performed the best in the in vivo tests. Upon examination of the SDS-PAGE gel PreTNA protein was not visible in contrast to MiTNA, PvTNA and EcTNA. Therefore, its higher productivity cannot be explained by larger amount of soluble enzyme available for Trp conversion. Apparently, as compared to in vivo, the biochemical conditions in the in vitro assay are less favorable for specifically the PreTNA enzyme.

**Table 1:** Kinetic constants of TNAs derived from *E. coli* (EcTNA), *M. indolicus* (MiTNA), *P. rettgeri* (PreTNA) *and P. vulgaris* (PvTNA)

TNA	K_M_ (Trp) [mM]	k_cat_ [s^−1^]	k_cat/_K_M_ [mM^−1^ s^−1^]
EcTNA	0.14 ± 0.01	1.65 ± 0.04	11.8 ± 3.5
MiTNA	0.29 ± 0.03	2.41 ± 0.06	8.3 ± 2.1
PreTNA	0.32 ± 0.04	0.46 ± 0.02	1.5 ± 0.4
PvTNA	0.03 ± 0.003	0.78 ± 0.02	26.0 ± 5.8

Kinetic constants were determined in triplicates, variation is shown as standard deviation

### Optimization of TNA-based indole production

Product loss in the later phase of cultivation was observed (Fig. 4). Since the off-gas analysis of the EcTNA-based bioreactor cultivation showed an indole loss at µg h^−1^ rates only, the product loss observed here was more likely due to biological rather than process-related reasons. The highest indole concentration was detected 24 h after inoculation of strain C1* (pGold-*PretnaA-CgaroP*), when glucose was not fully consumed. On the contrary, in culture supernatants of C1* expressing *EctnaA* the highest indole concentration and biomass formation were detected 24 h after inoculation and neither product loss nor further biomass formation were observed up to 48 h after inoculation (Fig. 4). It is therefore reasonable to assume that there is a correlation between product loss and growth. Reducing the carbon source concentration to 10 g L^−1^ (instead of 40 g L^−1^ glucose) limited biomass formation approximately to the concentration reached for highest indole concentration. C1*(pGold-*PretnaA-CgaroP*) did produce similar amounts of indole within 24 h and the product concentration remained stable during later phases of the cultivation (analysis of supernatants up to 48 h after inoculation). In a recent study, *C. glutamicum* strains were grown in presence or absence of indole and also in this study, decrease of indole over time was observed [31]. Interestingly, strains cultivated in presence of indole secreted Trp in late cultivation stage, while in control strains where no exogenous indole was added no Trp secretion was observed. This observation may possibly be due to the activity of the β-subunit of the tryptophan synthase, which catalyzes the condensation of indole with l-serine to liberate Trp as was already speculated by Walter and coworkers [31]. To gain a deeper understanding of the loss of indole late during the cultivation, further experimental characterization of the β-subunit of the tryptophan synthase, other enzymes acting promiscuously on indole and chemical instability are required.

Following the same rationale approach of reducing the carbon source concentration four fold, indole production was tested when the nitrogen concentration of the CGXII minimal medium was reduced four fold. This medium change increased indole accumulation corresponding to a Trp conversion of 89 mol-% (Table 2).

**Table 2:** Overview of indole production upon several optimization strategies

Strain	Media components				Indole determination (sampling time)	Trp conversion
	Carbon source	Nitrogen source	Trp	Indole		
C1*(pGold-*PretnaA-CgaroP*)	40 g L^−1^ glucose	20 g L^−1^ (NH_4_)_2_SO_4_ 5 g L^−1^ urea	1 g L^−1^	–	0.35 ± 0.01 g L^−1^ (24 h) 0.25 ± 0.01 g L^−1^ (48 h)	61 mol-% (24 h) 44 mol-% (48 h)
C1*(pGold-*PretnaA-CgaroP*)	40 g L^−1^ gluconate	20 g L^−1^ (NH_4_)_2_SO_4_ 5 g L^−1^ urea	1 g L^−1^	–	0.21 ± 0.01 g L^−1^ (24 h)	37 mol-%
C1*(pGold-*PretnaA-CgaroP*)	10 g L^−1^ glucose	20 g L^−1^ (NH_4_)_2_SO_4_ 5 g L^−1^ urea	1 g L^−1^	–	0.45 ± 0.01 g L^−1^ (24 h) 0.44 ± 0.01 g L^−1^ (48 h)	78 mol-% (24 h) 77 mol-% (48 h)
C1*(pGold-*PretnaA-CgaroP*)	10 g L^−1^ glucose	5 g L^−1^ (NH_4_)_2_SO_4_ 1.25 g L^−1^ urea	1 g L^−1^	–	0.51 ± 0.01 g L^−1^	89 mol-%
C1*(pGold-*PretnaA-CgaroP*)	10 g L^−1^ glucose	5 g L^−1^ (NH_4_)_2_SO_4_ 1.25 g L^−1^ urea	2 g L^−1^	–	0.77 ± 0.01 g L^−1^ (24 h) 0.91 ± 0.01 g L^−1^ (48 h)	67 mol-% (24 h) 79 mol-% (48 h)
C1*(pGold-*PretnaA-CgaroP*)	10 g L^−1^ glucose	5 g L^−1^ (NH_4_)_2_SO_4_ 1.25 g L^−1^ urea	2 g L^−1^ (0 h) 2 g L^−1^ (24 h)	–	0.94 ± 0.01 g L^−1^ (48 h)	41 mol-%
C1* *Δpyk* (pGold-*PretnaA-CgaroP)*	40 g L^−1^ glucose	20 g L^−1^ (NH_4_)_2_SO_4_ 5 g L^−1^ urea	1 g L^−1^	–	0.28 ± 0.01 g L^−1^	49 mol-%
C1* *Δpyk* (pGold-*PretnaA-CgaroP)*	40 g L^−1^ gluconate 1 g L^−1^ glucose	20 g L^−1^ (NH_4_)_2_SO_4_ 1.25 g L^−1^ urea	1 g L^−1^	–	0.18 ± 0.01 g L^−1^	31 mol-%
C1*(pGold-*PretnaA_*artRBS*-CgaroP*)	10 g L^−1^ glucose	5 g L^−1^ (NH_4_)_2_SO_4_ 1.25 g L^−1^ urea	1 g L^−1^	–	0.38 ± 0.01 g L^−1^	66 mol-%
C1*(pGold-*PretnaA-EcridA-CgaroP*)	10 g L^−1^ glucose	5 g L^−1^ (NH_4_)_2_SO_4_ 1.25 g L^−1^ urea	1 g L^−1^	–	0.37 ± 0.01 g L^−1^	66 mol-%
C1*(pGold-*PretnaA-EcyjgH-CgaroP*)	10 g L^−1^ glucose	5 g L^−1^ (NH_4_)_2_SO_4_ 1.25 g L^−1^ urea	1 g L^−1^	–	0.37 ± 0.01 g L^−1^	66 mol-%
IVO20 (pGold-*PretnaA-CgaroP*)	10 g L^−1^ glucose	5 g L^−1^ (NH_4_)_2_SO_4_ 1.25 g L^−1^ urea	2 g L^−1^ (0 h) 2 g L^−1^ (24 h)	–	0.68 ± 0.01 g L^−1^ (48 h)	30 mol-% (48 h)
IVO38 (pGold-*PretnaA-CgaroP*)	10 g L^−1^ glucose	5 g L^−1^ (NH_4_)_2_SO_4_ 1.25 g L^−1^ urea	2 g L^−1^ (0 h) 2 g L^−1^ (24 h)	–	0.63 ± 0.01 g L^−1^ (48 h)	27 mol-% (48 h)

When not specified otherwise, media components were added with inoculation and indole was determined in culture supernatants 24 h after inoculation

The Trp biosynthesis is strictly regulated in order to control its energetically expensive metabolism. Enzymes encoded by the *trp* operon are subject to feed-back inhibition by Trp [36, 37]. Moreover, transcription of the operon underlies tight control by an attenuator region upstream of the *trp* operon [38, 39]. When cultivated in minimal medium, supplementation of Trp is essential for indole production by the chassis strain C1*, however, Trp feeding also prevents de novo biosynthesis of the same. As consequence, cellular metabolism, such as the protein biosynthesis machinery, competes with TNAs for their common substrate Trp. Increasing TNAs activity in the cytosol of C1* was expected to increase the flux of Trp towards the designated product. Exchange of the standard ribosomal binding site upstream of *PretnaA* with an artificial one resulted in a more than two-fold increase of the TNA activity in the soluble protein fraction of *C. glutamicum* lysates (Table 3).

**Table 3:** TNA activity in soluble protein fraction of C1*

Strain	spec. TNA activity [mU mg^−1^]
C1*(pGold)	11 ± 2
C1*(pGold-*PretnaA-CgaroP*)	87 ± 3
C1*(pGold-*PretnaA_*artRBS*-CgaroP*)	210 ± 10

One unit (U) is defined as the amount of enzyme required to convert 1 µmol substrate within 1 min

However, under biotransformation conditions, the enhanced TNA activity did neither result in higher titers nor in faster indole accumulation. C1* strains carrying either the standard plasmid or the plasmid with artificial RBS upstream of *PretnaA* showed similar growth behavior and indole production profiles. Increased enzymatic activity did neither led to a higher conversion rate of Trp nor to a faster production of indole. Thus, complete conversion of the substrate remains pending.

### Flux enforcement as a strategy to increase indole production

The genome scale metabolic model of *C. glutamicum* ATCC 13032 [40] was adapted to represent the metabolism of the genome reduced C1* chassis strain. Model simulations showed indole production to be extremely limited at maximum growth conditions. Therefore, a flux-enforcement strategy to couple indole production to the biomass formation of C1* was applied in the next step. Production of amino acid (derivatives) such as lysine, 5-aminovalerate and glutarate had been successfully improved by coupling the production pathway to the central metabolism in *C. glutamicum* [41–43]. Since TNAs liberate pyruvate along with indole, the flux enforcement strategy was based on pyruvate biosynthesis. Therefore, a pyruvate bradytrophic strain is required. A major source of pyruvate in glucose catabolism is the glucose uptake itself, since *C. glutamicum* imports glucose via a phosphoenolpyruvate dependent transport system (PTS). Glucose uptake into the cell is coupled to phosphoenolpyruvate-dependent phosphorylation yielding glucose 6-phosphate and pyruvate. The genome-scale constrained-based model of C1* was used to sample and explore intracellular fluxes upon maximum growth. When gluconate (non-PTS uptake) serves as carbon source, these simulations identified pyruvate kinase as major pyruvate forming enzyme (92% of model predicted pyruvate amount), followed by pyruvate carboxylase (8% of model predicted pyruvate amount). Pyruvate formation from serine deaminase, malic enzyme 1 and 2 and anthranilate synthase was negligible during gluconate-dependent growth. Since it was shown that *C. glutamicum* lacking pyruvate kinase activity is not able to form biomass on non-PTS carbon sources such as ribose and maltose [44], an in-frame deletion of pyruvate kinase gene *pyk* in C1* was generated and transformed with pGold-*PretnaA-CgaroP*. As expected, C1*Δ*pyk* did not form biomass on the non-PTS substrates ribose and gluconate as sole carbon sources. Pyruvate release upon TNA-catalyzed Trp conversion was expected to subsequently initiate re-routing of the intracellular flux to accommodate intracellular Trp availability. Therefore, C1* *Δpyk (*pGold*-PretnaA-CgaroP*) was generated and cultivated in standard minimal medium supplemented with 40 g L^−1^ gluconate and 1 g L^−1^ Trp. Plasmid-based expression of *tnaA* with simultaneous feeding of Trp could not compensate for the absence of pyruvate kinase. Also, supplementation with 5 mM glucose could not initiate biomass formation on the non-PTS carbon sources (data not shown). It was observed that, while C1* *Δpyk (*pGold*-PretnaA-CgaroP*) did not form biomass when cultivated on the aforementioned non-PTS carbon sources, indole accumulated to 0.18 g L^−1^ in culture supernatants (Table 2). Clearly, the observed conversion of Trp to indole was not sufficient to bypass pyruvate bradytrophy and the strategy did not lead to flux-enforcement towards indole production. Efficient re-routing of the central carbon metabolism of the pyruvate bradytrophic strain upon Trp conversion may therefore require an adaptive laboratory evolution (ALE) approach. ALE was shown to be a powerful tool to improve re-routing of internal fluxes [45] in plethora of microbial hosts in order to select mutant strains with improved abilities such as higher growth rates, enhanced tolerance towards toxins or improved production properties [46].

### Relieving of indole toxicity results in increased indole production

Since the strategies described above did not improve indole production notably, inhibition of the conversion by potentially toxic compounds was considered. This could explain why strains with higher space-time yield also showed slower biomass formation, as was observed for strain C1*(pGold-*PretnaA-CgaroP*) when compared to C1*(pGold-*EctnaA-CgaroP*) (Fig. 4). Therefore, all cultivations described below were performed with strain C1*(pGold-*PretnaA-CgaroP*). The TNA reaction releases two potentially toxic compounds, indole itself and a short-lived enamine/imine intermediate [22]. Many PLP-dependent enzymes such as TNAs generate reactive enamine/imine intermediates, which spontaneously decompose to the respective ketones. In order to minimize damage by these metabolic compounds, several organisms carry reactive intermediate deaminases (Rid), which have been shown to accelerate the hydrolysis of enamine/imine to the less detrimental ketones [47, 48]. It was shown that RidA homologous prevent damage to PLP-dependent enzymes involved in different metabolic routes such as the branched chain amino acid synthesis [49] or glycine metabolism [50]. It is therefore tempting to speculate that accumulation of reactive intermediates of the TNA reaction in C1* causes cellular damage, which subsequently limits conversion of the substrate. Expression vectors carrying either *ridA* or *yjgH* (a *ridA* homolog) from *E. coli* in addition to *PretnaA* and *CgaroP* were constructed and used to transform C1*. Culture supernatants of strains co-expressing either *ridA* or *yjgH* did not show improved indole accumulation (Table 2). Since the overexpression of enzymes that detoxify enamine/imine intermediates did not improve indole production we assume that accumulation of enamine/imine intermediates is not critical for the process or could neither be avoided by RidA nor by YjgH.

By addition of 2 g L^−1^ Trp instead of 1 g L^−1^, indole accumulation increased from 0.5 ± 0.01 g L^−1^ to a titer of 0.8 ± 0.01 g L^−1^ within 24 h (Table 2). Further incubation of the cultures resulted in a final indole concentration of 0.9 g L^−1^. However, while the absolute indole concentration in culture supernatants increased, the molar conversion of the substrate decreased by about 10%. Moreover, the provision of up to 4 g L^−1^ Trp had no further impact on production. The highest indole concentration leveled at 0.9 g L^−1^ irrespective of adding 1, 2 or 4 g L^−1^ Trp and excess substrate remained in the medium. It is noteworthy, that indole accumulated to similar concentrations when 2 g L^−1^ Trp was added once at 0 h or twice at 0 and 24 h. Therefore, it appears that accumulation of the product in culture supernatants prevents complete conversion of the substrate (Table 2).

In in vivo studies of *C. glutamicum* ATCC 13032 and the chassis strain C1* performed by Walter et al., C1* showed higher tolerance to indole compared to its ancestor. The chassis strain reached comparable final OD_600_ values independent of indole exposure, but the strain took approximately three times longer to reach its final biomass concentration [31]. In the same study, tolerance of *C. glutamicum* ATCC 13032 towards indole could be improved by adaptive laboratory evolution to the level observed with the chassis strain C1*. Two evolved strains derived from the study of Walter et al. [31] were tested in combination with the pGold-*PretnaA-CgaroP* and showed even lower conversion rates compared to C1* (Table 2), indicating that improved tolerance towards indole in these strains was not beneficial for indole production from Trp.

Taken together, the consistent findings that the indole titer stagnated at about 0.9 g L^−1^ independent of the amount of substrate added indicates that the negative effects of indole itself on the fitness of the cell are likely to be limiting the biotransformation process. Exposure of the chassis strain C1* to 8 mM (0.9 g L^−1^) indole perturbed growth severely as was shown recently [31].

One strategy to circumvent product toxicity is in situ product recovery (ISPR) by which the product concentration in the aqueous phase is maintained at sub-inhibitory concentrations. The liquid-liquid interaction by direct addition of a solvent has been used in several processes for extraction of aqueous insoluble or toxic compounds from *C. glutamicum* cultures and was therefore the method of choice. Addition of dodecane to *C. glutamicum* cultures improved biotechnological production of terpenoids such as pinene [51], patchoulol [52] and valencene [53]. Further, comparative transcriptome analysis of cultures subjected to a dodecane overlay revealed only minor effects of the solvent to the host [53]. The aroma compound o-methyl anthranilate was produced by *E. coli* and *C. glutamicum* strains with overlays of tributyrin [29]. A recent study investigated selection criteria for food-grade solvents preferred in microbial flavor and fragrance production. Aromatic molecules such as vanillin and phenyl acetate were efficiently extracted by the esters dibutyl maleate and/or dibutyl sebacate [54]. When applying ISPR in a bioconversion process, the solvent of choice must not only efficiently extract indole from the aqueous phase, but Trp must remain in the medium in order to be available for uptake into the host. Therefore, extraction capacities of the five different hydrophobic solvents dodecane, triacetin, tributyrin, dibutyl maleate, and dibutyl sebacate using aqueous solutions of indole and Trp were determined. Dodecane showed the lowest extraction capacity of all tested solvents (85%), while the four esters showed high indole extraction capacity with values between 97 and 99% (Additional file 1: Fig. S4A). In addition, these solvents showed low Trp extraction capacity (5 to 15%) (Additional file 1: Fig. S4B), and thus, proved to be suitable for further production tests in a growth-coupled biotransformation. Bioconversion tests of C1* (pGold-*PretnaA-CgaroP*) in the adapted CGXII medium applying ISPR with dibutyl sebacate and tributyrin was executed. Analysis of both phases showed accumulation of indole in the solvent phase, while only traces remained in the aqueous medium phase. After normalizing with respect to the different volumes of the organic and aqueous phases and the different molecular weights, complete molar conversion of the substrate was revealed as 2 g L^−1^ Trp was converted to 1.2 g L^−1^ indole 9 h after inoculation. This translated to a space-time yield of 0.13 g L^−1^ h^−1^.

**Fig. 5:**
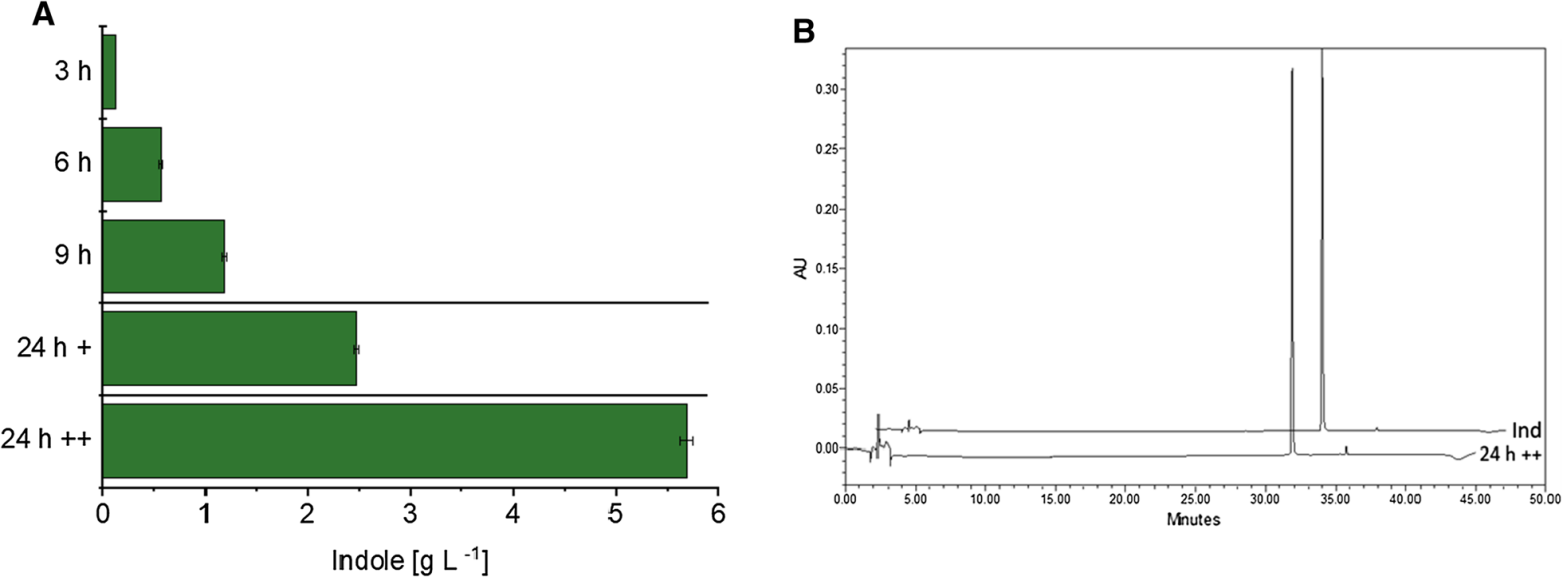
In situ product recovery enabled production of indole in g per liter scale. C1*(pGold-*PretnaA-CgaroP*) was cultivated in CGXII supplemented with 10 g L^−1^ glucose, 2 g L^−1^ Trp and 20% (v/v) dibutyl sebacate. Production titer at 3 h, 6 h and 9 h is shown. 24 h+: indole accumulation in dibutyl sebacate, when additional 2 g L^−1^ Trp were supplemented 9 h after inoculation. 24 h ++: indole accumulation in dibutyl sebacate, when additional 8 g L^−1^ Trp were supplemented 9 h after inoculation. Indole concentrations were determined in solvent layer by HPLC exclusively, since aqueous phase was not enriched by the same. Indole concentrations were normalized to the volume of the medium phase, as they were 5-times concentrated in the overlay phase. Means and arithmetic errors of duplicate cultures are shown. **A** HPLC chromatograms of the overlay of the cultivation 24 h ++ recorded at 270 nm and of an indole standard (Ind) are shown (**B**)

Since the overlay was not saturated with indole, the impact of repeated addition of Trp on indole production was tested. Indeed, a second dose of 2 g L^−1^ or 8 g L^−1^ Trp added 8–9 h after inoculation led to indole titers of 2.5 g L^−1^ and 5.7 g L^−1^, respectively (Fig. 5A).

These titers observed 24 h after inoculation indicated complete molar conversion of Trp to indole within this time period. Thus, ISPR allowed full conversion of up to 10 g L^−1^ supplemented Trp and increased the space-time-yield by one order of magnitude from 0.02 g L^−1^ h^−1^ (0.9 g L^−1^ indole within 48 h; Table 2) to 0.24 g L^−1^ h^−1^. Chromatographic analysis of the hydrophobic solvent overlay showed high purity of indole (Fig. 5B), paving the way to a downstream process for efficient biotechnological production of natural indole. Establishment of a biotechnological TNA-based process for indole production of almost 6 g L^−1^ provides an attractive route for fragrance and flavor applications. Yet, for industrial application, further optimization could include optimization of medium to organic solvent ratio and process automatization in a (fed-batch) bioreactor cultivation. The latter one may also benefit distribution of dibutyl sebacate in the medium and thus, improve product extraction.

Production of indole by exogenous tryptophan addition was previously described by the natural indole producer *E. coli.* Similar to observations made here, indole production in presence of up to 10 mM Trp (2 g L^−1^) did not exceed 5–6 mM (0.6–0.7 g L^−1^). The study showed that the amount of exogenous tryptophan, not the amount of TNA, determined the final amount of indole accumulation [25]. A recent study described complete conversion of 10 mM Trp by *E. coli* [55], however, indole did not accumulate as it was only an intermediate that was converted to the insoluble indigo by oxidation and subsequent dimerization. Also conjugation of indole to glucose by oxidation and subsequent glycosylation proved suitable for production of indole derivatives in a gram scale [56]. Thus, other approaches to avoid accumulation of free indole by its functionalization or conjugation to sugars have been suitable to avoid its toxicity.

## Conclusion

Heterologous expression of known and newly selected *tnaAs* in the food-grade production host *C. glutamicum* enabled formation of indole upon Trp supplementation. In vivo tests of several TNA candidates in the host organism *C. glutamicum* showed that a candidate from *P. rettgeri* (PreTNA) supported indole production best. This holds true for both the reaction rate and the maximum product yield achieved. The major bottleneck of indole production from Trp, i.e., the negatively effects on the cell physiology and, consequently, the transformation efficiency was overcome by applying different kinds of food-grade solvents. Complete molar conversion was achieved and indole production titers of 5.7 g L^−1^ were reached within 24 h. In this way a new sustainable process for indole production for the fragrance and flavor industry was established.

## Experimental

### Bioprospecting of TNA genes

A non-redundant list of protein sequences of bacterial origin annotated as TNAs were retrieved from BioCyc (https://biocyc.org/) in March 2019. The list was built by searching for co-occurrence of the term ‘tryptophanase’ and the EC number 4.1.99.1. The initial list with over 900 sequences was reduced to 407 by considering a single representative sequence per species. Pairwise alignment and percentage identify matrix among amino acid sequences were computed using Clustal Omega [57]. Hierarchical clustering was performed using 1-similarity as a distance metric and the R hclust command; the tree was cut so that 14 clusters were identified using the R cut-tree command. Heatmaps were built using the *pheatmap* R package (https://CRAN.R-project.org/package=pheatmap).

### Modelling

An existing genome scale metabolic model of *C. glutamicum* ATCC 13032 [40] was adapted to the C1* strain. Reactions in the model associated to genes deleted in the C1* strain, were removed from the model. Reaction identification was done by considering the gene-protein-reaction associations in the original model. The obtained model was verified to simulate growth on a broad range of carbon sources namely glucose, acetate, maltose, fructose, gluconate, sucrose, d-arabinol and pyruvate achieving the same in silico growth rates as the originating model. A reaction representing indole production from Trp was added to the model to simulate the effect of the TNA, an indole exchange reaction was added to the model to enable production simulations. Model simulations were run by setting the lower bounds of all media components representing salts and minerals to − 1000 mmol gDW^−1^ h^−1^ to simulate unlimited availability. Similar, lower bound of the oxygen exchange reaction were set to − 1000 mmol gDW^−1^ h^−1^ to simulate aerobic growth. The lower bound of the reaction corresponding to uptake of the selected carbon source (glucose) was limited to − 10 mmol gDW^−1^ h^−1^ to limit maximum uptake. Trp addition to the medium was simulated by setting the lower bound of the corresponding exchange reaction to − 10 mmol gDW^−1^ h^−1^. Model simulations were run using flux balance analysis by setting as objective either the biomass synthesis reaction (to simulate maximum growth conditions) or the indole exchange reaction, to simulate indole production. Sampling of the solution space was performed using artificial centering hit-and-run with 10,000 sampled points. All model simulations were performed using python (v3.8) and cobrapy (v0.22) [58].

### Strains and growth conditions

All strains and plasmids used in this study are listed in Tables 4 and 5.

**Table 4:** Bacterial strains used in this study

Strain	Description	Source
WT	*C. glutamicum* wild type, ATCC13032	American Type Culture Collection
C1*	Genome reduced chassis strain derived from WT	[32]
C1* Δ*pyk*	C1* with in-frame deletion of pyruvate kinase gene *pyk*	This work
IVO20	Strain evolved from WT in the presence of indole after 20 transfers	[31]
IVO38	Strain evolved from WT in the presence of indole after 38 transfers	[31]

**Table 5:** Vectors used in this study

Strain	Description	Source
pGold	Km^R^, P_trc_*lacI*^q^, pGA1, *oriV*_*Ec*_; *C. glutamicum/E. coli* expression shuttle vector with *Bsa*I recognition site for Golden Gate assembly	[30]
pK19*mobsacB*	Km^R^, *E. coli*/*C. glutamicum* shuttle vector for construction of insertion and deletion mutants in *C. glutamicum* (pK18 *oriVEc sacB lacZα*)	[59]
pACYCDuet-1	Cm^R^, *P15A* (pACYC184 replicon), encodes two multiple cloning sites, each of which is preceded by a T7 promoter, a lac operator, and a ribosome binding site	Novagen
pGold-*EctnaA*	pGold expressing *tnaA* from *E. coli* MG1655	This work
pGold-*EctnaA-EctnaB*	pGold expressing *tnaA* and *tnaB* from *E. coli* MG1655	This work
pGold-*EctnaA-CgaroP*	pGold expressing *tnaA* from *E. coli* MG1655 and *aroP* from *C. glutamicum* ATCC13032	This work
pGold-*HstnaA*	pGold expressing *tnaA* from *Histophilus somni*	This work
pGold-*MitnaA*	pGold expressing *tnaA* from *Mageeibacillus indolicus*	This work
pGold-*SstnaA*	pGold expressing *tnaA* from *Synechocystis *sp.	This work
pGold-*PrtnaA*	pGold expressing *tnaA* from *Proteiniclasticum ruminis*	This work
pGold-Cx*tnaA*	pGold expressing *tnaA* from *Corynebacterium xerosis*	This work
pGold-*EhtnaA*	pGold expressing *tnaA* from *Escherichia hermannii*	This work
pGold-*CbtnaA*	pGold expressing *tnaA* from *Clostridiales bacterium*	This work
pGold-*PretnaA*	pGold expressing *tnaA* from *Providencia rettgeri*	This work
pGold-*PptnaA*	pGold expressing *tnaA* from *Prevotella pallens*	This work
pGold-*SmtnaA*	pGold expressing *tnaA* from *Sciscionella marina*	This work
pGold-*NstnaA*	pGold expressing *tnaA* from *Nocardioides *sp.	This work
pGold-*RstnaA*	pGold expressing *tnaA* from *Rhodobacter sphaeroides*	This work
pGold-*PstnaA*	pGold expressing *tnaA* from *Photobacterium *sp.	This work
pGold-*PvtnaA*	pGold expressing *tnaA* from *Proteus vulgaris*	This work
pGold-*HstnaA-CgaroP*	pGold expressing *tnaA* from *Histophilus somni* and *aroP* from *C. glutamicum* ATCC13032	This work
pGold-*MitnaA-CgaroP*	pGold expressing *tnaA* from *Mageeibacillus indolicus* and *aroP* from *C. glutamicum* ATCC13032	This work
pGold-*PrtnaA-CgaroP*	pGold expressing *tnaA* from *Proteiniclasticum ruminis* and *aroP* from *C. glutamicum* ATCC13032	This work
pGold-*PretnaA-CgaroP*	pGold expressing *tnaA* from *Providencia rettgeri* and *aroP* from *C. glutamicum* ATCC13032	This work
pGold-*PptnaA-CgaroP*	pGold expressing *tnaA* from *Prevotella pallens* and *aroP* from *C. glutamicum* ATCC13032	This work
pGold-*PvtnaA-CgaroP*	pGold expressing *tnaA* from *Proteus vulgaris* and *aroP* from *C. glutamicum* ATCC13032	This work
pGold-*PretnaA_artRBS-CgaroP*	pGold expressing *tnaA* from *Providencia rettgeri* with artificial RBS and *aroP* from *C. glutamicum* ATCC13032	This work
pGold-*PretnaA-EcridA-CgaroP*	pGold expressing *tnaA* from *Providencia rettgeri* with artificial RBS, *ridA* from *E. coli* MG1655 and *aroP* from *C. glutamicum* ATCC13032	This work
pGold-*PretnaA-EcridA-CgaroP*	pGold expressing *tnaA* from *Providencia rettgeri* with artificial RBS, *yjgH* from *E. coli* MG1655 and *aroP* from *C. glutamicum* ATCC13032	This work
pK19- Δ*pyk*	pK19*mobsacB* derivative with the flanking region of *pyk*	[44]
pACYCDuet-*EctnaA*	pACYCDuet-1 expressing *tnaA* from *E. coli* MG1655	This work
pACYCDuet-*MitnaA*	pACYCDuet-1 expressing *tnaA* from *Mageeibacillus indolicus*	This work
pACYCDuet-*PretnaA*	pACYCDuet-1 expressing *tnaA* from *Providencia rettgeri*	This work
pACYCDuet-*PvtnaA*	pACYCDuet-1 expressing *tnaA* from *Proteus vulgaris*	This work

Cloning was performed using *E. coli* DH5α [60], *E. coli* BL21(DE3) was used for gene expression for protein purification. Fermentative production was performed using the chassis strain *C. glutamicum* C1* [32]. Single colonies of *E. coli* strains from a fresh Lysogeny Broth (LB; 10 g L^−1^ tryptone, 10 g L^−1^ sodium chloride and 5 g L^−1^ yeast extract) agar plate were inoculated for overnight cultures in LB in flasks (10% filling level) grown at 37 °C and 250 rpm. Single colonies of *C. glutamicum* strains were inoculated in 50 mL Brain-Heart-Infusion (BHI) in 500 mL baffled flasks at 30 °C and 180 rpm for overnight cultures. When necessary, the medium was supplemented with kanamycin (25 µg mL^−1^), or chloramphenicol (25 µg mL^−1^). The gene expression from the expression vectors pGold and pACYCDuet-1 was induced by addition of 1 mM isopropyl-β-d-1-thiogalactopyranoside (IPTG). For fermentative production, *C. glutamicum* cells were incubated in BHI overnight on a rotary shaker, harvested (4000×*g*, 5 min) and washed once with TN buffer (50 mm TrisHCl, 50 mm NaCl, pH 6.3). The cells were inoculated to an optical density at 600 nm (OD_600_) of 1 in 50 mL CGXII minimal medium [61] supplemented with the indicated amount of carbon source. Growth in 500 mL baffled flasks was followed by measuring the OD_600_ using V-1200 Spectrophotometer (VWR, Radnor, PA, USA).

### Bioreactor cultivation

Fermentation of *C. glutamicum* C1* was performed in an initial working volume of 0.75 L in a bioreactor (Sartorius, 1 L vessel equipped with baffles) at 30 °C, and an aeration rate of 1 slpm. The vessel was equipped with two Rushton turbines, one for mixture of the culture and the second blade was installed 2 cm above the volume height. The stirrer speed was controlled to maintain the relative dissolved oxygen saturation at 30%. The pH was maintained at 7.0 by controlled addition of KOH (4 M) and phosphoric acid (10% (v/v)). The antifoam Struktol® J647 was added manually when necessary to avoid foaming. Samples were taken when indicated, immediately harvested and stored at – 20 °C until analysis. For bioreactor cultivation a modified CGXII minimal medium was used lacking the MOPS component: 20 g L^−1^ (NH_4_)_2_SO_4_, 5 g L^−1^ urea, 1 g L^−1^ K_2_HPO_4_, 1 g L^−1^ KH_2_PO_4_, 40 g L^−1^ glucose in addition to the same concentrations of trace elements and vitamins as described elsewhere [61]. The bioreactor was inoculated to an OD_600_ of 1 by addition of a flask culture grown in standard CGXII minimal medium.

### Molecular genetic techniques and strain construction

The standard molecular genetic techniques were performed as described in Green and Sambrook [62]. Transformation of *E. coli* DH5α was performed by heat shock [62], plasmid DNA transfer into *C. glutamicum* by electroporation [61]. The pGold constructs for expression in *C glutamicum* were constructed applying the Golden Gate cloning strategy [63] with *Bsa*I as type IIS restriction enzyme (New England Biolabs (NEB), Bioké, Leiden, The Netherlands). The vector pGold was predigested with *Bsa*I and treated with alkaline phosphatase (NEB) for higher cloning efficiency. The gene sequences of all used *tnaA* genes were codon optimized for expression in *C. glutamicum* and ordered with the respective overhang for Golden-Gate cloning at GenScript (Piscataway, New Jersey, USA). *TnaB* from *E. coli* and *aroP* from *C. glutamicum* were amplified from the respective genome using the respective primer (Table 6) and the Q5 polymerase (NEB) according to the protocol.

**Table 6:** Oligonucleotides used in this study

Oligonucleotide	Sequence 5′-> 3′
*EctnaB*_fw	GGTCTCTGCAACTAGGAGGATTACAAAATGACTGATCAAGCTGAAAAA
*EctnaB*_rv	GGTCTCAATACTTAGCCAAATTTAGGTAACACG
*CgaroP*_fw	GGTCTCTGCAACTAGGAGGATTACAAAATGGCTAAATCTAATGAAGGG
*CgaroP*_rv	GGTCTCAATACTCAGTTCAAGTCGGAAGGG
*EcridA*_fw	cctgcaggtcgactctagagTATTAATAAGGAGGTAACATGAGCAAAACTATCGCGAC
*EcridA*_rv	attcgagctcggtacccgggTTAGCGACGAACAGCGATC
*EcyjgH*_fw	cctgcaggtcgactctagagATCATAAAGGAGGTATATTTATGGTAGAAAGAACCGCTG
*EcyjgH*_rv	attcgagctcggtacccgggTTACTGCTCAGGGATGCG
PretnaA_artRBS_fw	gaGGTCTCTCAGATGGCCGTAGCTTAAGGAGGTATAGTATGGCAAAGCGCATCGTTG
PretnaA_rv	tccGGTCTCATTGCTTACTTGATTGGCTTCAGACG

Cloning of the pACYCDuet-1 expression vectors encoding for *tnaA* with N-terminal 6xHis tag was performed by restriction with BamHI (NEB) and NotI (NEB) and ligation by T4 ligase (NEB). The TNA genes were amplified using the respective primer pair for addition of the restriction sites using Phusion polymerase (Fisher Scientific, Landsmeer, The Netherlands) according to the protocol. Correct integration of the genes into expression vectors was verified by colony PCR and subsequent sequencing (Macrogen Europe, Amsterdam, The Netherlands). All primers used are shown in Table 6. The pGold plasmids were used to transform *C. glutamicum* strains while pACYCDuet-1 plasmids were used to transform *E. coli* BL21(DE3).

### Gene expression and isolation of recombinant protein for *in vitro* characterization

For expression, 5 mL overnight cultures of the recombinant *E. coli* BL21(DE3) strains were prepared. Main cultures in 100 mL 2xTY (16 g L^−1^ Tryptone, 10 g L^−1^ yeast extract, 5 g L^−1^ sodium chloride) with 25 mg mL^−1^ chloramphenicol in a 500 mL baffled flask were inoculated to an OD_600_ of 0.05 from the overnight cultures and inoculated at 37 °C and 180 rpm. When an OD_600_ of 0.6 was reached, gene expression was induced by addition of 1 mM isopropyl-β-d-thiogalactopyranoside (IPTG) and the cultures were transferred to 18 °C and 180 rpm. The cells were harvested (2740×*g*, 10 min, 4 °C) after 22 h incubation, washed once with 100 mM KPi pH 7.8 and stored at − 20 °C for further analysis.

For protein purification, cell pellets were resuspended in 5 mL lysis buffer (50 mM NaH_2_PO_4_, 300 mM NaCl, 10 mM imidazole, 1 mg mL^−1^ lysozyme and 1% (v/v) protease inhibitor cocktail and incubated on ice for 30 min. Cells were disrupted by sonication (on ice, 10 × 10 s with 10 s break, MSE Soniprep 150, amplitude 14) and insoluble particles were removed by centrifugation (20,200×*g*, 30 min, 4 °C). The soluble protein fraction was purified by affinity chromatography using Ni-NTA. For column preparation, 300 µL of NiNTA beads were added to a filter column. The stationary phase was equilibrated using 1 mL washing buffer (50 mM NaH_2_PO_4_, 300 mM NaCl, 20 mM imidazole) and the soluble protein fraction was added to the column. The stationary phase was washed 5x with 1 mL washing buffer and his-tagged proteins were eluted by addition of 5 × 150 µL elution buffer (50 mM NaH_2_PO_4_, 300 mM NaCl, 300 mM imidazole) and incubation for 5 min before elution. Each fraction was collected separately and analyzed on an acrylamide gel. Pure elution fractions were pooled and buffer was exchanged to storage buffer (50 mM NaH_2_PO_4_, 300 mM NaCl, 12.5% (v/v) Glycerol and 1 mM dithiothreitol) using ultrafiltration concentrators with a cutoff of 10 kDa. Aliquots of purified protein were stored at − 20 °C for further analysis.

### TNA enzymatic assay

To determine the enzymatic kinetics of the TNA, an activity assay based on assays described by Kiick and Phillips 1988 and Nuidate et al., 2015 was combined and performed [64, 65]. Based on the fact that TNA convert Trp to indole, pyruvate and ammonium, the readout system was established by reduction of pyruvate to lactate catalyzed by a NADH-dependent lactate dehydrogenase as indicator reaction. In this coupled assay, one molecule Trp is required to produce one molecule lactate under oxidation of one molecule of NADH.

The enzyme kinetic was followed by colorimetric change of the NADH absorbance at a wavelength of 340 nm. The assay was performed in a micro-titer plate reader (Bio-Rad Laboratories, Hercules, California, US) using 96-well plates (PP, Greiner) at 30 °C. In a 100 mM KP_i_ buffer pH 7.8, 1 mM dithiothreitol, 0.05 mM pyridoxal phosphate, 0.2 mM NADH, and 100 U mL^−1^ lactate dehydrogenase from rabbit muscle (Merck, Darmstadt, Germany) and a suitable amount of the purified TNA were mixed. The reaction was started by addition of the substrate Trp. For determination of the Michaelis constants (K_M_), various concentrations ranging from 6.25 µM to 10 mM Trp were added. The kinetic parameters of the substrate affinity (K_M_) and the maximum activity (v_max_) were determined using Origin with the add-on ‘Enzyme kinetics’. Specific enzymatic activity is given in units, where one unit (U) is defined as the amount of enzyme required to convert 1 µmol substrate within 1 min.

### Quantification of aromatic compounds

Extracellular aromatic compounds were quantified by high-performance liquid chromatography (HPLC) (e2695, Waters, Milford, Massachusetts, USA). Culture samples were collected at different time points and centrifuged (20,200×*g*, 15 min, 4 °C) to separate biomass and culture supernatant. One volume of culture supernatant was mixed with one volume of ultrapure water and two volumes of methanol, sonicated for 15 min and centrifuged (20,200×*g*, 15 min, 4 °C). Separation was performed by a reversed phase HPLC using a RP18 column (Luna RP18 3µ; 100 A 150 × 2 mm, Phenomenex) equipped with two pre-columns, a flow rate of 0.19 mL min^−1^ and an injection volume of 5 µL. The following gradient of solvent A (0.1% (v/v) formic acid) and solvent B (acetonitrile + 0.1% (v/v) formic acid) was applied as mobile phase: 0–25 min a linear gradient of 5–35% B, 25–27 min a linear gradient of 35–75% B, 27–32 min 75% B, 32–34 min a linear gradient of 75–5% B, 34–40 min 5% B. Detection was performed with a photo diode array detector (2998, Waters, Milford, Massachusetts, USA) at 270 nm for indole and 280 nm for Trp. In order to analyze indole captured in tributyrin and dibutyl sebacate the gradient of solvent A and B was adjusted to the following: 0–25 min a linear gradient of 5–35% B, 25–27 min a linear gradient of 35–90% B, 27–37 min 90% B, 37–39 min a linear gradient of 90–5% B, 39–45 min 5% B.

For GC-MS analysis, nonpolar compounds were extracted from 4 mL culture supernatants by addition of 2 mL ethyl acetate, vigorous shaking followed by centrifugation (1200×*g*, 10 min, 4 °C). The ethyl acetate layer was transferred to a fresh glass tube and washed twice with ultrapure water to remove salts. The clear ethyl acetate phase was dried over sodium sulphate and subsequently used for analysis. Samples were analyzed by GC-MS according to published literature [31].

For analysis of the loss of indole in the bioreactor off-gas, tubes equipped with Tenax, a hydrophobic matrix, were connected to the off-gas tubes for exactly 15 min. Subsequent Thermo desorption-MS analysis was performed as described elsewhere [66].

## Supplementary Information

**Additional file 1. Fig. S1: **Indole production of C1* (pGold-EctnaA-CgaroP) in bioreactor cultivation. **Fig. S2**. Exploration of sequence identity between TNA sequences grouped in 14 clusters. **Fig. S3**. Acrylamide gel of the soluble protein fraction of C1* expressing different TNA genes. **Fig. S4**. Partitioning of indole (A) and Trp (B) between water and the respective solvent.

**Additional file 2**. Codon optimised sequences of TNA genes optimized for expression in *C. glutamicum* used in this study. 

## Data Availability

All data generated or analyzed during this study are included in this published article and its additional files.
