# Supplementary material for: Production of indole by Corynebacterium glutamicum microbial cell factories for flavor and fragrance applications
Source: Microb Cell Fact. 2022 Mar 24;21:45. doi: 10.1186/s12934-022-01771-y (PMC8944080; doi:10.1186/s12934-022-01771-y)
Supplement: Supplementary file 1 — Additional file 1. Fig. S1: Indole production of C1* (pGold-EctnaA-CgaroP) in bioreactor cultivation. Fig. S2. Exploration of sequence identity between TNA sequences grouped in 14 clusters. Fig. S3. Acrylamide gel of the soluble protein fraction of C1* expressing different TNA genes. Fig. S4. Partitioning of indole (A) and Trp (B) between water and the respective solvent. [file 12934_2022_1771_MOESM1_ESM.docx]

Additional file 1 to

**Production of indole by *Corynebacterium glutamicum* microbial cell factories for flavor and fragrance applications**

**Melanie Mindt^a,b^, Arman Beyraghdar Kashkooli^a,^*, Maria Suarez-Diez^c^, Lenny Ferrer^d^, Tatjana Jilg^d^, Dirk Bosch^a^, Vitor Martins dos Santos^c,e^, Volker F. Wendisch^d^ and Katarina Cankar^a,#^**

^a^ Business Unit Bioscience, Wageningen Plant Research, Wageningen University & Research, Wageningen, The Netherlands

^b^ Axxence Aromatic GmbH, Emmerich am Rhein, Germany

^c^ Laboratory of Systems and Synthetic Biology, Wageningen University & Research, Wageningen, The Netherlands

^d^ Genetics of Prokaryotes, Faculty of Biology & CeBiTec, Bielefeld University, Bielefeld, Germany

^e^ Laboratory of Bioprocess Engineering, Wageningen University & Research, Wageningen, The Netherlands

^#^ Corresponding author: Katarina Cankar, [katarina.cankar@wur.nl](mailto:katarina.cankar@wur.nl)

* Current address: Department of Horticultural Science, Faculty of Agriculture, Tarbiat Modares University

**
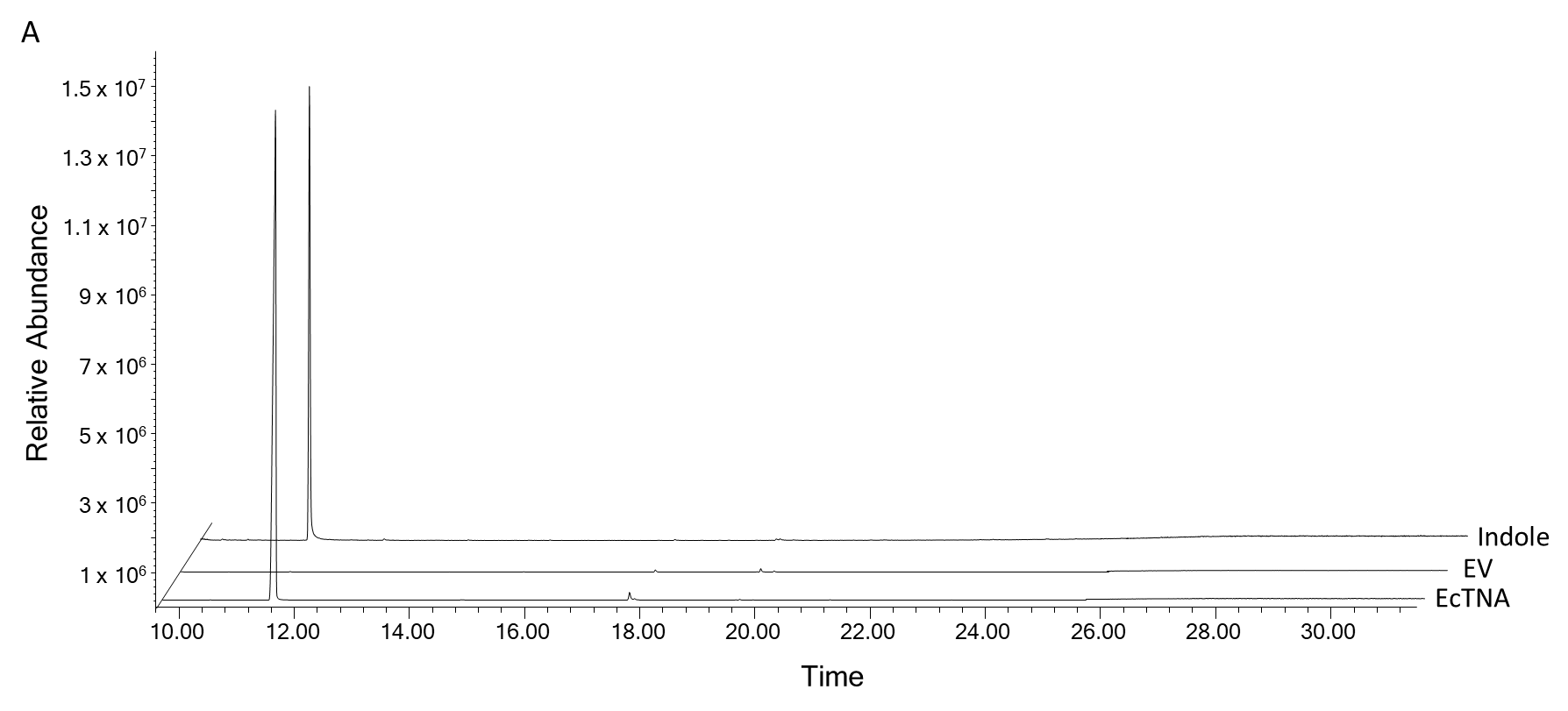
**

**
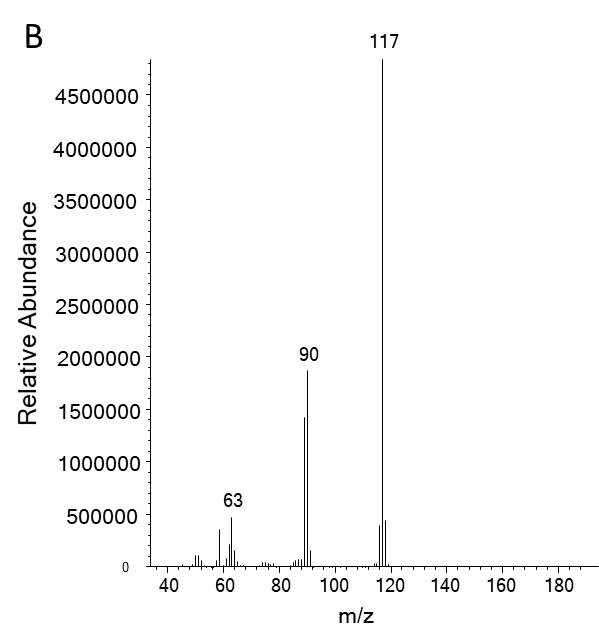

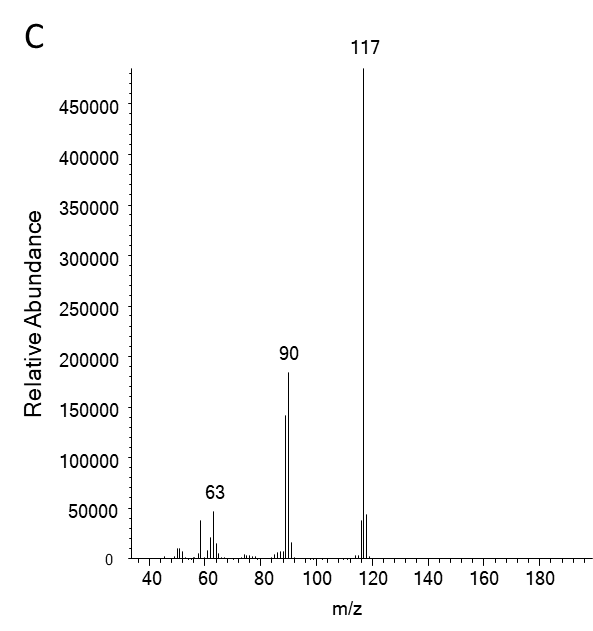
**

**Fig. S1:** Indole production of C1* (pGold-*EctnaA-CgaroP*) in bioreactor cultivation. GC-MS chromatograms of hydrophobic extracts of culture supernatants (A). Mass spectra of indole from EcTNA sample (B) and indole standard (C), which also correspond to the respective entry of NIST mass spectrometry data center. EcTNA: C1* (pGold-*EctnaA-CgaroP*); EV: C1* (pGold).


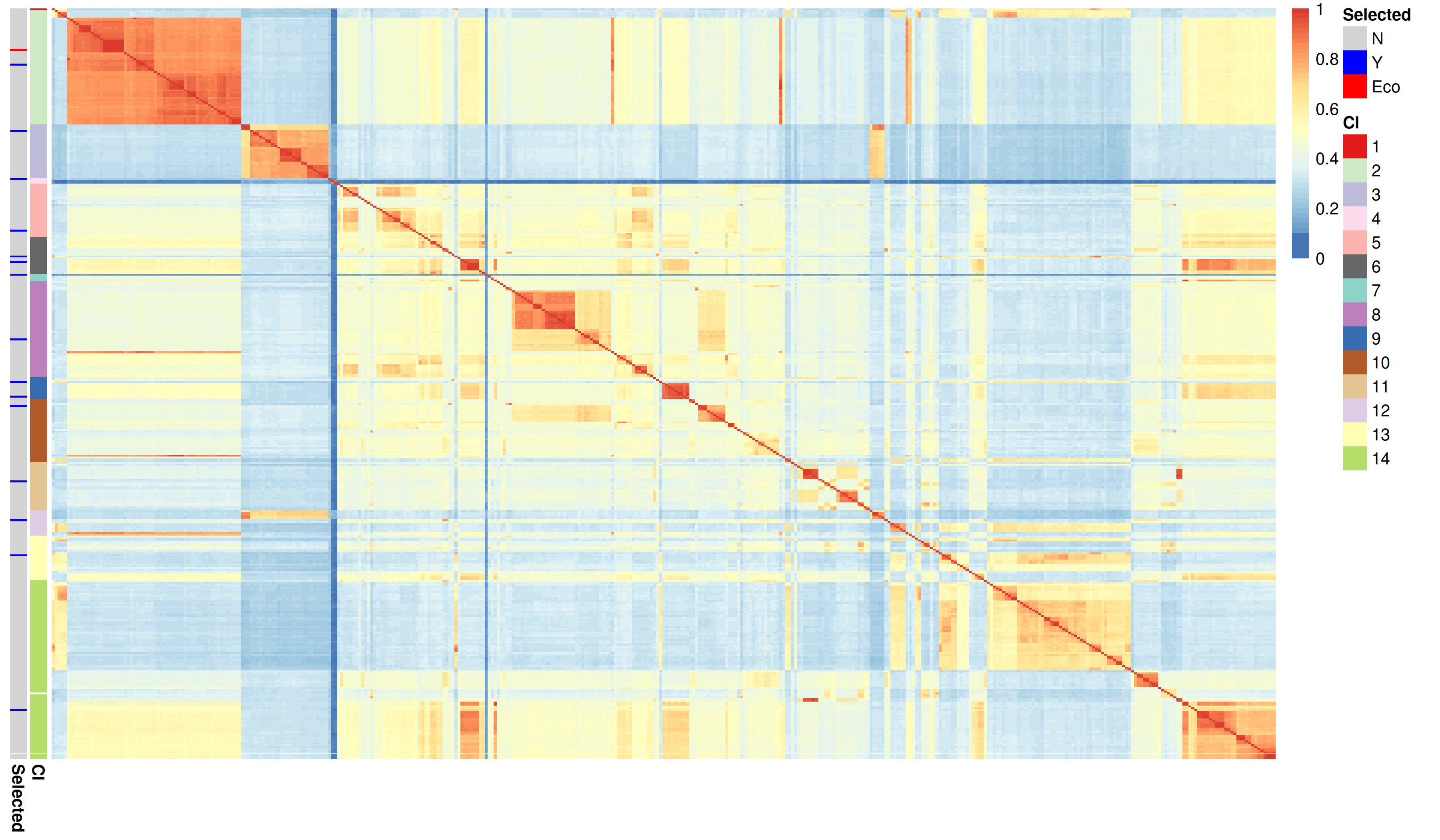


**Fig. S2.** Exploration of sequence identity between TNA sequences grouped in 14 clusters. Red color of the heatmap indicates high identity and the blue color indicates low identity between protein sequences. The 14 clusters are indicated in different colors on the left hand side of the heatmap (column Cl). Selected candidates from each cluster are indicated on the left side of the heatmap (column Selected) with a blue line and the *E. coli* TNA (Eco) is indicated by a red line.


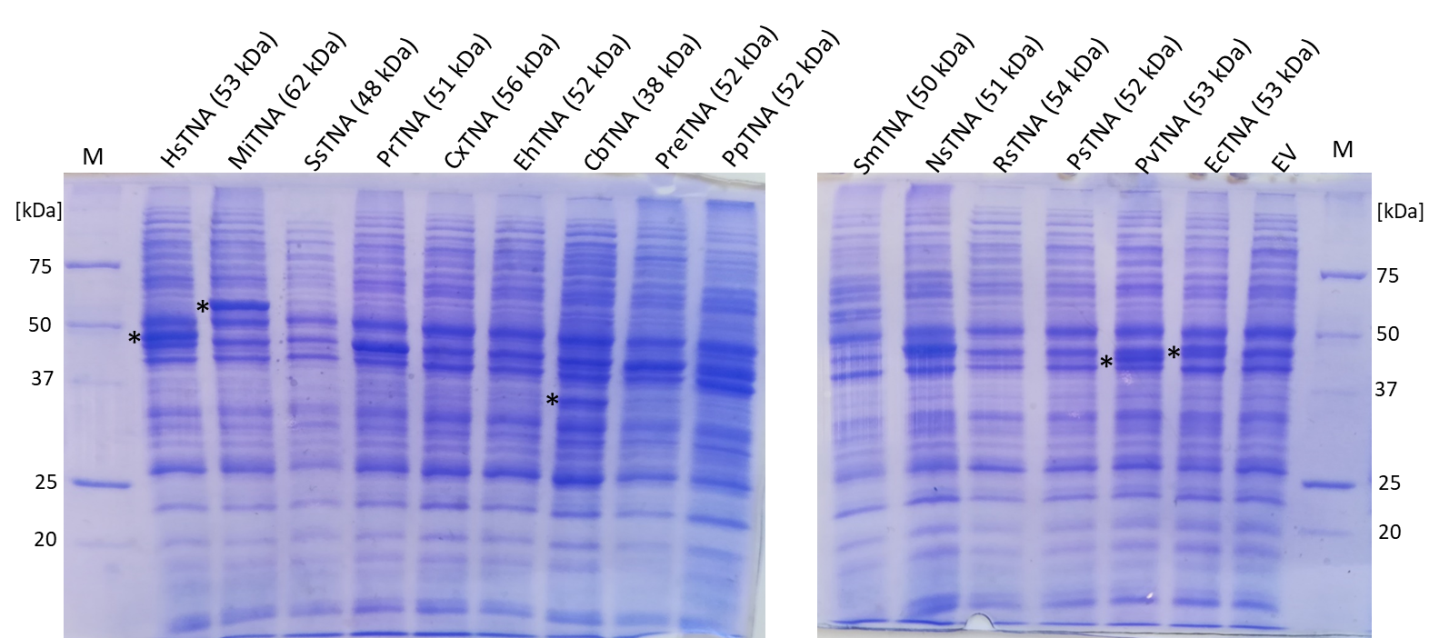


**Fig. S3**. Acrylamide gel of the soluble protein fraction of C1* expressing different TNA genes. The theoretical size of the respective TNA is shown in parentheses. Visible protein bands correlating to the expected size are marked with an asterisk.


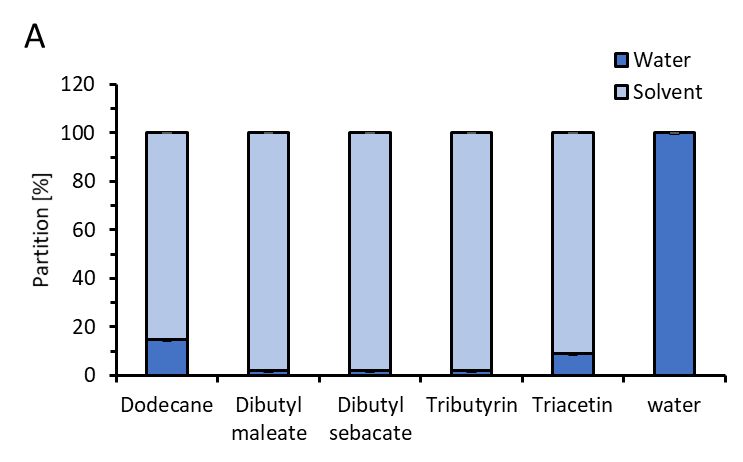


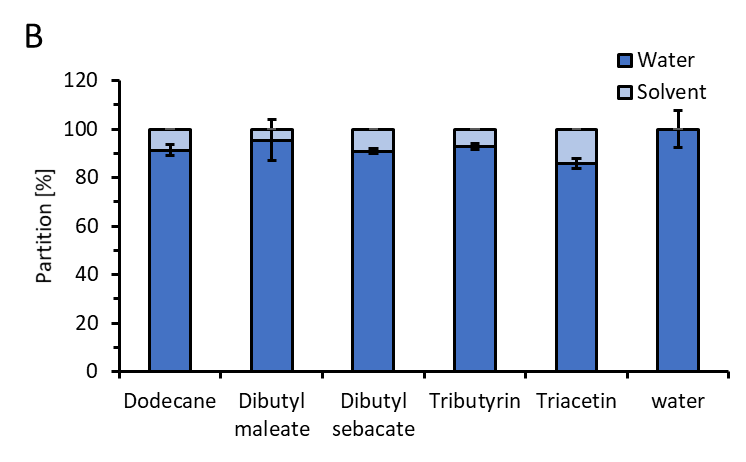


**Fig. S4.** Partitioning of indole (A) and Trp (B) between water and the respective solvent. The compounds were dissolved in water to a concentration of 2 g L^-1^. 10 % (v/v) of the respective solvent was added and incubated at 30°C for 60 min under rigorous shaking. Remaining indole or Trp was analyzed by HPLC in aqueous phase. Determination are shown as average from triplicate samples with standard deviation.
