# Supplementary material for: Production of indole by Corynebacterium glutamicum microbial cell factories for flavor and fragrance applications
Source: Microb Cell Fact. 2022 Mar 24;21:45. doi: 10.1186/s12934-022-01771-y (PMC8944080; doi:10.1186/s12934-022-01771-y)
Supplement: Supplementary file 2 — Additional file 2. Codon optimised sequences of TNA genes optimized for expression in C. glutamicum used in this study. [file 12934_2022_1771_MOESM2_ESM.docx]

Additional file 2 to

**Production of indole by *Corynebacterium glutamicum*** **microbial cell factories for flavor and fragrance applications**

**Melanie Mindt^a,b^, Arman Beyraghdar Kashkooli^a,^*, Maria Suarez-Diez^c^, Lenny Ferrer^d^, Tatjana Jilg^d^, Dirk Bosch^a^, Vitor Martins dos Santos^c,e^, Volker F. Wendisch^d^ and Katarina Cankar^a,#^**

^a^ Business Unit Bioscience, Wageningen Plant Research, Wageningen University & Research, Wageningen, The Netherlands

^b^ Axxence Aromatic GmbH, Emmerich am Rhein, Germany

^c^ Laboratory of Systems and Synthetic Biology, Wageningen University & Research, Wageningen, The Netherlands

^d^ Genetics of Prokaryotes, Faculty of Biology & CeBiTec, Bielefeld University, Bielefeld, Germany

^e^ Laboratory of Bioprocess Engineering, Wageningen University & Research, Wageningen, The Netherlands

^#^ Corresponding author: Katarina Cankar, [katarina.cankar@wur.nl](mailto:katarina.cankar@wur.nl)

* Current address: Department of Horticultural Science, Faculty of Agriculture, Tarbiat Modares University

**Codon optimised sequences of TNA genes optimized for expression in *C. glutamicum* used in this study**

TNA candidate genes were ordered at GenScript as synthetic sequences codon optimized for expression in *C. glutamicum* including but not limited to the following criteria:

- Codon usage bias
- GC content
- CpG dinucleotides content
- mRNA secondary structure
- Cryptic splicing sites
- Premature PolyA sites
- Internal chi sites and ribosomal binding sites
- Negative CpG islands
- RNA instability motif (ARE)
- Repeat sequences (direct repeat, reverse repeat, and Dyad repeat)
- Restriction sites that may interfere with cloning

The following sequences have been used for *in vivo* characterization:

NP_418164.4_*Escherichia-coli*_EcTNA_CO

ATGGAAAACT TCAAGCACCT GCCAGAGCCA TTCCGCATCC GCGTTATCGA ACCAGTGAAG 60

CGTACCACCC GTGCTTACCG TGAAGAGGCC ATCATCAAGT CCGGCATGAA CCCATTCCTG 120

CTGGATTCCG AGGACGTGTT CATCGATCTG CTGACCGACT CCGGCACCGG TGCTGTCACC 180

CAGTCCATGC AGGCAGCTAT GATGCGCGGC GACGAAGCAT ACTCCGGTTC CCGCTCCTAC 240

TACGCACTGG CTGAGTCCGT CAAGAACATC TTCGGCTACC AGTACACCAT CCCAACCCAC 300

CAGGGTCGCG GTGCTGAACA GATCTACATC CCAGTCCTGA TCAAGAAGCG CGAACAGGAG 360

AAGGGTCTGG ACCGCTCCAA GATGGTTGCC TTCTCCAACT ACTTCTTCGA TACCACCCAG 420

GGCCACTCCC AGATCAACGG TTGCACCGTG CGCAACGTCT ACATCAAGGA AGCATTCGAT 480

ACCGGCGTGC GCTACGACTT CAAGGGTAAC TTCGATCTGG AAGGCCTGGA GCGCGGTATC 540

GAAGAGGTCG GCCCAAACAA CGTCCCATAC ATCGTTGCCA CCATCACCTC TAACTCCGCA 600

GGCGGTCAGC CAGTTTCCCT GGCTAACCTG AAGGCCATGT ACTCCATCGC TAAGAAGTAC 660

GACATCCCAG TGGTCATGGA TTCCGCCCGC TTCGCAGAAA ACGCTTACTT CATCAAGCAG 720

CGCGAAGCAG AGTACAAGGA CTGGACCATC GAACAGATCA CCCGCGAAAC CTACAAGTAC 780

GCAGACATGC TGGCCATGTC CGCAAAGAAG GATGCTATGG TTCCAATGGG CGGTCTGCTG 840

TGCATGAAGG ATGACTCCTT CTTCGATGTG TACACCGAAT GCCGCACCCT GTGCGTTGTG 900

CAGGAAGGCT TCCCAACCTA CGGCGGTCTG GAAGGCGGTG CAATGGAGCG CCTGGCTGTT 960

GGCCTGTACG ATGGTATGAA CCTGGACTGG CTGGCATACC GCATCGCTCA GGTGCAGTAC 1020

CTGGTCGATG GCCTGGAAGA GATCGGTGTT GTTTGCCAGC AGGCTGGCGG TCACGCAGCA 1080

TTCGTGGATG CTGGCAAGCT GCTGCCACAC ATCCCAGCAG ATCAGTTCCC AGCCCAGGCA 1140

CTGGCTTGCG AACTGTACAA GGTTGCTGGC ATCCGCGCAG TGGAGATCGG CTCCTTCCTG 1200

CTGGGTCGCG ACCCAAAGAC CGGCAAGCAG CTGCCATGCC CAGCTGAACT CCTGCGTCTG 1260

ACCATCCCAC GTGCAACCTA CACCCAGACC CACATGGATT TCATCATCGA AGCTTTCAAG 1320

CACGTGAAGG AGAACGCTGC CAACATCAAG GGCCTGACCT TCACCTACGA ACCAAAGGTC 1380

CTGCGCCACT TCACCGCAAA GCTGAAGGAA GTGTAA 1416

WP_075293819.1*_Histophilus-somni_*HsTNA_CO

ATGGAAAACT TCGAGCACCT GCCAGAACCA TTCCGCATCC GCGTTATCGA ACCAGTGAAG 60

CGTACCACCC GTGCATACCG CGATGAATCC ATCCTGAAGG CAGGCATGAA CCTGTTCCTG 120

CTGGATTCCG AGGACATCTT CATCGATCTG CTGACCGACT CCGGCACCGG TGCTGTTACC 180

CAGGATATGC AGGCAGCTAT GCTGCGCGGC GACGAAGCAT ACTCCGGTTC CCGCTCCTAC 240

TACGTGCTGG CTAACGCCGT CAAGGAAATC TTCGGCTACG AGTACACCAT CCCAACCCAC 300

CAGGGTCGCG GTGCTGAGCA GATCTACATC CCAGTGCTGA TCAAGAAGCG CGAACAGGAG 360

AAGGGTCTGG ATCGCAACAA GATGGTGGTG TTCTCCAACT ACTTCTTCGA CACCACCCAG 420

GGCCACTCCC AGCTGAACGG TGCAACCGTG CGCAACGTCT ACATCAAGGA AGCTTTCGAT 480

ACCGACGTCG ATCACGACTT CAAGGGCAAC TTCGATCTGG AAAAGCTGGA GCAGGGCATC 540

CTGGAAGTGG GTGCAAACAA CGTCCCATAC ATCGTTTGCA CCATCACCTG CAACTCCGCT 600

GGCGGTCAGC CAGTTTCCCT GGCAAACATG AAGGCTATGT ACCAGATCGC CCGCAAGTAC 660

GATATCCCAG TGATCATGGA CTCCGCACGC TTCGCTGAAA ACGCCTACTT CATCCAGCAG 720

CGCGAAGCAG AGTACAAGGA CTGGACCATC GAACAGATCA CCTACGAGTC CTACAAGTAC 780

GCAGATGCTC TGGCCATGTC CGCCAAGAAG GACGCAATGG TCCCAATGGG CGGTCTGCTG 840

TGCTTCAAGG ATAACTCTAT GGAAGATGTT TACAACGAGT GCCGCACCCT GTGCGTTGTG 900

CAGGAAGGCT TCCCAACCTA CGGCGGTCTG GAAGGCGGTG CTATGGAGCG TCTGGCTGTT 960

GGCCTGCGCG ATGGTATGCG CCAGGACTGG CTGGCTTACC GCATCTCCCA GATCGAATAC 1020

CTGGTGCAGG GCCTGGAGAA GATCGGTGTT GTTTGCCAGC AGCCAGGCGG TCACGCAGCA 1080

TTCGTCGATG CTGGCAAGCT GCTGCCACAC ATCCCAGCCG AACAGTTCCC AGCACAGGCT 1140

CTGGCCTGCG AGCTGTACAA GGTTGCAGGC ATCCGCTCCG TGGAAATCGG CTCCCTGCTG 1200

CTGGGTCGCG ACCCAAAGAC CGGTCAGCAG CTGCCATGCC CAGCTGAGCT GCTGCGTCTG 1260

ACCATCCCAC GTGCAACCTA CACCCAGACC CACATGGATT TCATCATCGA AGCATTCAAG 1320

CAGGTGAAGG AGAACGCTAA CAACATCAAG GGCCTGGACT TCACCTACGA ACCAAAGGTC 1380

CTGCGCCACT TCACCGCACG CCTGAAGGAG ATCTAA 1416

WP_012992776.1_*Mageeibacillus-indolicus_*MiTNA_CO

ATGTCCAAGA ACTACCCACT GAACGTGCCA ACCCCACACC ACTTCACCTT CGCAGTCCGC 60

GACCTGCCAA ACGTCACCGT TGAACAGCGC GAGCGTGTGC TGCGTGCTAC CCACTACAAC 120

GAGTTCGCCT TCCCAGCTGG TATGCTGACC GTCGATATGC TGTCCGACTC CGGCACCACC 180

GCAATGACCA ACCAGCAGTG GGCTTCCCTG TTCCTGGGCG ACGAAGCATA CGGCCGCAAC 240

ACCGGTTACT ACGTGCTGCT GGATACCTTC CGCGACATCT TCGAACGCGG CGGTGAGAAG 300

CACTGGAAGA AGATCCTGGA TCTGGTGCGC ACCGATTGCC GCGACGTCGA AAAGATGATG 360

GACGAGGTTT ACCTGTGCGA ATACGATGGC GGTCTGTTCA ACGGCGGTGC AGCTCAGATG 420

GAGCGCCCAA ACGCATTCAT CATCCAGCAG GGTCGTGCTG CAGAGTCCGT GCTGATGGAA 480

ATCGTCCGCA ACATCCTGGC TAAGCGCCAC CCAGGCAAGG TTTTCACCAT CCCATCCAAC 540

GGTCACTTCG ACACCACCGA AGGCAACATC AAGCAGATGG GTTCCATCCC ACGCAACCTG 600

TACAACAAGA AGCTGCTGTA CGAAGTGCCA GAGGGCGGTC ACTACGCCAA GAACCCATTC 660

AAGGGTAACA TGGATATCGA AAAGCTGGAG CAGCTGATCC ACGCAGTTGG CCCAGAGAAC 720

GTGCCACTGA TCTTCACCTG CATCACCAAC AACCCAGTCT GCGGTCAGCC AGTGTCTATG 780

GCAAACATCC GCGAAATCAA CAAGGTGGCC CACAAGTACA ACATCCCACT GGTTTTCGAC 840

GTGGCTCGCT GGGCCGAGAA CTGCTACTTC ATCAAGATGA ACGAAGATGG CTACGCTGAC 900

AAGTCCATCG CAGAAATCGC TACCGAGATG TTCTCCTACT GCGACGCTTT CTGCATGTCC 960

GCCAAGAAGG ATGGTCACGC CAACATGGGC GGTATGATGG CATTCCGCGA CAAGGGCCTG 1020

TTCTGGAAGA ACTTCTCCGA TTTCAACCCA GACGGCTCCA TCAAGACCGA TGTGGGTGTC 1080

CTGCTGAAGG TCAAGCAGAT CTCCTGCTAC GGCAACGACT CCTACGGCGG TATGTCCGGT 1140

CGCGATATCA TGGCACTGGC TGTCGGCCTG TACGAGTCCT GCAACTTCGA TTACCTGCAC 1200

GAGCGCGTTG GCCAGGCAGA ATACCTGGCT CAGGGTTTCT ACAAGGCCGG CGTTAAGGGT 1260

GTGGTCCTGC CAGCCGGCGG TCACGCAGTG TACATCAACA TGGATGAATT CTTCGACGGC 1320

AAGCGCGATC ACACCACCTT CGCTGGCGAG GGTTTCTCCC TGGAACTGAT CCGCCGCTAC 1380

GGCATCCGCG TCTCCGAGCT GGGTGACTAC TCTATGGAAT ACGATCTCAA GACCCCAGAA 1440

CAGCAGGCCG AGCTGGCAAA CGTTGTGCGC TTCGCTATCG ACCGCTCCCG CCTGACCCAG 1500

GAGCACCTGG ATTACGTCAT CGCTGCCGTT AAGGCCCTGT ACGAAGATCG CGAGTCCATC 1560

CCAAACATGC GCATCGTTTG GGGCCACAAC CTGCCAATGC GCCACTTCCA CGCATTCCTG 1620

GAACCATACG CTCCAAAGGC CTAA 1644

WP_009630937.1*_Synechocystis-sp._*SsTNA_CO

ATGAACCTGA CCGAAGCTCG CTCCCACCGC ATGCAGGTTC TGGAGCTGGC CGGCCACAAC 60

CTGGATATCA TCCCATCCGA AAAGATCACC CTGGATCTGC AGTCCGACTC CCTGATGCAC 120

AAGTCCCTGC CACCAGTGGG CTACGGTTCC GAATTCCACC AGGAGCGCTG CCACTCCAAC 180

ATCTCCGTCG AAGATATCTT CTCCCGCTAC TTCGGCTTCC CATACGTCAT GGCCGTTTCC 240

CAGGGTCGCC TGGCTGAGGC CATCCTGTCC CACGCTACCA TCCGCAACGG CCACTACATC 300

CCAGGTTCCT CCCTGTTCCC AACCACCAAG GTGCACCAGG AACGCAACGG CGCAACCCCA 360

GTTGAAGTGA TGTCCGCCGA GTCCCTGGAT GTTGCATCCC CATACCCATT CAAGGGTAAC 420

ATCGACATCG CAGCTCTGGA GCAGGTGATC CAGACCTACC ACCCACGCTG GATTCCATAC 480

ATCTGCGTCG AACCATGCAA CAACGCAGTT GGCGGTCACC CAATCTCCCT GGAGAACATG 540

CGCGCAGTGG CTGATCTGGC TCACCACTAC CACATCCCAG TCTACCTGGA TGCTTGCCGC 600

ATCATCGACA ACGCCTACCT GATCCAGGAA CGCGAGGACC AGTACCGCAA CACCCCAGTC 660

GGCGAAATCA TCCGCGAGTT CTGCTCCTAC GCAGATGGTT GCACCATGTC CGCTACCAAG 720

GACTTCCCAA CCTCTATCGG CGGTTTCTTC GCAACCCGCG ATCCAGAGCT GTTCTACCGT 780

TGCGTGGATC AGGTTGCACT GCTGGGCTCC GGTCTGTCCC ACGTCGCAAA GGAAAACCTG 840

GCCTACGCAA TGAACAACAT GGATGACGTT TTCGAGCGCG TGCGCAAGCG CATGAACATC 900

GTTAAGAAGC TGCACGATGC TCTGCAGGCA CACCCAGTGG TTGCACGCCC AGCTGGCGGT 960

CACGCAATCT TCCTGAACGC TTCCACCCAG AACCTGGGCA TCCCACACCA GCTGCACCCA 1020

GAACAGGCTT TCCTGCACCG CCTGTTCTCC GACTACGGCA TCCGCGGTTC CGTGAACCAC 1080

TCCTCCCCAG CCCAGATCGC AAACAACATC TCCTTCGTTC GCTTCGCACT GCCAATCATG 1140

GGCCTGTCCG TGGAAGAGAT CGCTAAGGCC GCAGATGACA TCTCCGCTGT CCTGGCCGAT 1200

AAGGACTCCA TCCAGGGTCT GGAGATCGTT AAGAAGTACC CAGGCCTGAC CGGTTTCATG 1260

CGCTCCCACT ACCGCCCAGT CACCTAA 1287

WP_031577100*_Proteiniclasticum-ruminis_*PrTNA_CO

ATGTCCGTCA AGTACTTCGC TGAACCATTC AAGATCAAGA TGGTTGAGCC AATCAAGATC 60

CTGACCCGCG AAGAGCGCCT GAAGAAGATC CAGGAAGCAA ACTACAACGT GTTCTCCCTG 120

GCAGCTGAGG ATTGCTACAT CGACCTGCTG ACCGATTCCG GCACCGGTGC CATGTCCGCA 180

GACCAGTGGG CTGGCATCAT GCGCGGTGAT GAATCCTACT CCGGCTCCAA ATCCTTCTAC 240

CGCCTGCAGG AGTCCGCCAA GGACATCTTC GGCTACACCT TCATCCAGCC AGTTCACCAG 300

GGTCGCGCCG CAGAAAAGGT GGTCATGCCA ATCCTGCTGG GCGAGGGCAA GGTGTCCATC 360

TCCAACATGC ACTTCGACAC CACCCGCGCT CACGTTGAAA TCGCAGGTGC TCGTGCTGTG 420

GACTGCGTTG TGCCAGAAGC ACTGGATACC GAGTCCTACG CTCCATTCAA GGGTAACATG 480

GATAACGAAC GCCTGGTCCG CCTGATCGAA GAGTACGGCA AGGAGAACGT GGGTGCAATC 540

ATCATCACCG TCACCAACAA CTCCGCTGGC GGTCAGCCAG TTTCCATGAA GAACATCAAG 600

GAAACCTCTG AGATCGCCAA GCGCTTCGGC ATCAAGGTCG TTATCGACGC TGCCCGCTTC 660

GCAGAAAACT GCCACTTCAT CAAGCGTCGC GAAGAGGGCT ACGGTGATAA GTCCATCCGC 720

GAAATCGCCC GCGAGCTGTT CTCCTACGGC GACCTGTTCA CCATGTCCTC CAAGAAGGAT 780

GCAATCGTGA ACATCGGCGG TCTGGTGGGT GTCAAGGAAG ATGCCGACCT GTTCGAGAAG 840

GTCAAGGCAA ACACCATCCC ATTCGAGGGC TTCATCTCCT ACGGCGGTCT GGCTGGTCGC 900

GACCTGGAAG CACTGGCTAT CGGCCTGCAG GAAGGCGTGG AAGAGGATTT CCTGAAGTAC 960

CGCATCGGCC AGATGGAATA CCTGTCCTCC AAGCTGACCG AGGCTGGTAT CCCACACCAG 1020

ACCCCAGCTG GCGGTCACGC CATCTTCCTG GACGCCAAGA AGCTGCTGCC ACACATCCCA 1080

TACCACGAGT TCCCAGGCCA GGCTCTGACC ATCGAACTGT ACCTGGAGGC CGGCATCCGC 1140

GGTTGCGATA TCGGCTCCTA CATGCTGGGT AACGATCCAG ACACCGGCGT CCAGCTGGAA 1200

TCCCAGTTCG AGTTCACCCG CCTGGCAGTC CCACGTCGCG TTTACACCCA GTCCCACCTG 1260

GACGTTATCG TGGAAGCTCT GATCGAGATC AAGAAGCGCA AGGATTCCCT GAAGGGCTAC 1320

GAAATCACCT GGGAGCCAAA GATCCTGCGC CACTTCACCG CAAAGCTGCA GCCACTGAAG 1380

TAA 1383

WP_046649605.1*_Corynebacterium-xerosis_*CxTNA_CO

ATGGTTTCCG TGCCAGCAAA CGTTTCCATC CGCCTGTGCA AGGGTGCTCC AGATGTGTCC 60

AAGGTCAAGT TCTACCGCGG CGAGCAGCTG CCACTGGAAA TCCACAAGGT GCGCATCATC 120

CAGAAGCTGA CCCTGCTGCC AATCGAGCAG CGTCGCGAAG CAATGGAAGA GGCTGGTTTC 180

AACACCTTCC TGCTGCAGAA CGCCGATGTG TTCCTGGACA TGCTGACCGA TTCCGGCGTC 240

AACGCAATGT CCCAGGACCA GCAGGCAGCT ATGCTGATGG CCGATGACGC ATACGCTGGC 300

TCCGCAACCT ACACCCGCCT GTACGACAAG CTGGTTGAGA TCTTCGGTAT GGATTACTTC 360

CTGCCAGCAC ACCAGGGTCG TGCAGCAGAA AACATCATCT CCCAGACCAT GATCCGCCCA 420

GGCACCCTGA TCCCAATGAA CTACCACTTC ACCACCACCA AGCAGCACAT CACCGTCAAC 480

GGCGGTGAGG TGGTCGAACT GATCCGCCCA GAGGGTCTGG AAGTCACCTC TGACCACCCA 540

TTCAAGGGCA ACTTCGATGT TGAGGCTCTG CGCGGTCTGA TCGATGAGCG CGGCGCAGAA 600

GCAATCTCCC ACGTTCGCAT GGAAGCCGGC ACCAACCTGA TCGGCGGTCA GCCATTCTCC 660

CTGGAGAACC TGCGCGAAGT GGCTGCTACC TGCCGCGAGC ACTCCCTGCC ACTGGTCCTG 720

GATGCATCCC TGCTGGCAGA TAACCTGCAC TTCATCAAGA CCCGCGAAGA GGCATGCAAG 780

GACATGTCCA TCCGCGAGAT CACCCGCGCT ATGGCCGATG AAGTCGACGT TCTGTACTTC 840

TCCGCTCGTA AGCTCGGTTT CGGTCGCGGC GGTGGCATCT GCATCCGTGA TGAAGAAACC 900

TTCAAGAAGA TGCGCGGTTA CGTGCCAATG TTCGAGGGCT TCCTGACCTA CGGTGGCATG 960

TCCGTTCGCG AGATGGAAGC TATCACCGTG GGTCTGGACG AAACTATGGA TGAAGATATG 1020

ATCAACCAGG GCCCACAGTT CATCGAGTAC ATGGTTGATG AACTGGACCG TCGCAACATC 1080

CCAGTGATCA CCCCAGCAGG TGGCCTGGGT GCTCACGTTG ATGCAATGTC CTTCGTTGAT 1140

CACGTGCCAC AGAACGAATA CCCAGCAGCT GCCCTGGCAG CTGCCCTCTA CGTTGCTTCC 1200

GGTGTCCGTG GTATGGAGCG TGGCACTATG GCAGAACAGC GCGATGCTGA CGGCAACGAG 1260

CCACTGGCTG ATATGGAACT GGTCCGCCTG GCCATGCCAC GTCGCGTTTT CACCCTGTCC 1320

CAGGTCAACT ACGTTATCGA TCGCCTGGAC TGGCTGTACC AGAACCGCCG CCTGATCGGT 1380

GGCATGGAGT GGGAAGAGGA ACCAGAAATC CTGCGCTTCT TCTACGGCCG CCTCAAGACC 1440

AAGGGTAACT GGATGGGCGA CCTGGTCGAA CAGTTCCGCA AGGATTTCGG CGACTCCCTG 1500

TAA 1503

WP_002436225.1*_Escherichia-hermannii_*EhTNA_CO

ATGAAGCGCA TCCCAGAGCC ATTCCGCATC AAGATGGTTG AAAACATCCG CATGACCACC 60

TTCGATGACC GCGTGAAGGC ACTGGAAGAG GCTGGCTACA ACCCATTCCT GCTGAAGTCC 120

GAAGATGTGT ACATCGACCT GCTGACCGAT TCCGGCACCG GTGCAATGTC CGATCGCCAG 180

TGGGCTGGCC TGATGATGGG TGACGAGGCT TACGCCGGCT CCCGCAACTA CTTCAACCTG 240

TGCGAAAAGG TCCAGGAGAT CATCGGTTAC CCATACACCA TCCCAACCCA CCAGGGTCGC 300

GGTGCTGAAC AGATCCTGTT CCCATCCCTG ATCGCCCGCT GCAAGTCCAA GCGCCCAGTG 360

TTCATCTCCA ACTTCCACTT CGATACCACC GCAGCTCACG TTGAGCTGAA CGGCGCAAAG 420

GCTATCAACG TGGTCACCCC AAAGGCATTC GACACCACCT CTTGGTACGA TTGGAAGGGC 480

AACTTCGACA TCCCACAGCT GAAGGCTACC ATCGCCGAAC ACGGTGCTGA GAACGTCGCC 540

GCAATCATCA CCACCGTTAC CTGCAACTCC TCCGGCGGTC AGCCAGTGTC CCTGGCCAAC 600

ATGCGCGAAG TCTACGAGAT CGCAAAGCAG CACCACATCC CAGTTGTGAT CGATTCCGCT 660

CGCTTCTGCG AAAACGCCTG GTTCATCAAG CAGCGCGAAG AGGGCTACTC CAACAAGACC 720

GTTAAGGAAA TCATCCGCGA GATGTACCAG TACGGTGATA TGCTGACCAT GTCCGCAAAG 780

AAGGACCCAC TGGTGAACAT CGGCGGTCTG TGCTGCTTCC GCGATGACGA GGACCTGTTC 840

AACGAAGTGC GCATCCGCTG CGTCCCAATG GAGGGTTTCG TCACCTACGG CGGTCTGGCA 900

GGTCGTGATA TGGAGGCCCT GGCAATCGGC CTGGAAGAAG GCACCAACGA AGATTTCCTG 960

GCTTACCGCA TCAACCAGGT TGAATACCTG GGTGAACGCC TGCGCGAAGG CGGTATCCCA 1020

ATCCAGTACC CAACCGGCGG TCACGCCGTG TTCGTTGACG CAAAGCTGCT GCTGCCACAC 1080

ATCCCAGCAG AGCAGTTCCC AGCTCACGCC CTGAACAACG AGCTGTACCT GGAAGCTGGC 1140

ATCCGCTCCG TTGAAATCGG CTCCCTGCTG CTGGGTCGCG ATCCAGAAAC CGGCGAGCAG 1200

AAGCCATCCC CAATGGAGCT GCTGCGCCTG ACCATCCCAC GTCGCGTGTA CACCAACGAT 1260

CACATGGACT ACATCGCAGA CGCTCTGATC GCAGTCAAGG CCCGCGCATC CTCCATCAAG 1320

GGCCTGACCT TCACCTACGA ACCACCAGTT CTGCGCCACT TCGTGGCTCG CCTGAAGCCA 1380

GTCAACTAA 1389

ZP_04671823.1*_Clostridiales-bacterium*_CbTNA_CO

ATGTACTGGA TCGATCTGCG CTCCGACACC GTGACCCAGC CAACTATGGC AATGCGCCAG 60

GCAATGAAGG ATGCTATCGT GGGTGATGAC GTCTACGGCG ATGACCCAAC CGTCAACCAG 120

CTGGAAACTA TGGCAGCTCG TCGCATGGGC AAGGAAGCCG CACTGTTCGT GGTGTCCGGC 180

ACTATGGGCA ACCAGCTGGC TGTTATGACC CACACCACCC CAGGCCAGGA GATCATCGCC 240

AACCGCAACT GCCACATCAT CCACTACGAA TGCGGCTCCC CAGCACGCCT CTCCGGTGTG 300

GGTTACGCAC TGACCGATCG TGAGGACGGC ACCGTTACCG CAGAAGATGT GATCTCCCTG 360

AAGCGCCCAG AGCACGATGC ACACTTCCCA GCAACCGGTC TGGTCTGCGT TGAAAACGCC 420

CTGTGCAACG GCACCGTTGT GCCAATGGAC GTCCTGCGTC GCACCTGCGG CACCGCTCAC 480

GATCACCACA TCCCAGTTCA CCTGGACGGT GCACGCATCT TCAACGCTGC CCTGGCTCTG 540

GGCGTTGATG CAGCTGAGAT CGCCGCATGC GCAGACTCCG TGATGTTCTG CATCTCCAAG 600

GGCCTGTGCG CTCCAGTCGG TTCCCTGCTG TGCGGCACCA AGGATTTCAT CGAAAAGGCA 660

CGTGCTAACC GTAAGGCACT GGGCGGTGGC ATGCGCCAGG CAGGTGTCAT CGCTGCATGC 720

GGCGTTCTGG CCCTGGAAGA TATGGTCGAG CGCCTGAAGG AAGATCACGA CAACGCAAAG 780

TACCTGGGCG AGCGCCTGAA CGAAATCCCA GGCATCTACG CTGATATGGA CCGCATCCAG 840

ATCAACATGG TGTTCTGGAA AACCGATATC AAGGGTTTCA CCTCTTACGG CTTCGTGGAT 900

TACATGGACA AGAAGGGCGC TAAGGTCTAC GGTATCCTGG GCGACGAGTA CCGCTTCGTT 960

ACCTCTCACG ATACCCCACG CCGCTCCCTG GACCAGGTGA TCCGTCTGAT CCAGGAATAC 1020

ATCCACACCC TGTAA 1035

WP_042846291.1_*Providencia-rettgeri_*PreTNA_CO

ATGGCAAAGC GCATCGTTGA ACCATTCCGC ATCAAGATGG TGGAGAACAT CCGCATCCCA 60

TCCCGCGAAG AGCGCGAAGC AGCTCTGAAG GAAGCAGGTT ACAACCCATT CCTGCTGCCA 120

TCCTCCGCTG TGTACATCGA TCTGCTGACC GACTCCGGCA CCAACGCTAT GTCCGATCAC 180

CAGTGGGCCG CAATGATCAC CGGCGACGAA GCCTACGCAG GTTCCCGCAA CTACTACGAT 240

CTGAAGGACA AGGTGAAGGA GATGTTCGAT TACGACTACG TCATCCCAGC ACACCAGGGT 300

CGCGGTGCTG AAAACATGCT GTTCCCAGTC CTGCTGAAGG TTAAGCAGGA GCAGGGCGGT 360

GCAAAGAAGC CAGTGTTCAT CTCCAACTTC CACTTCGATA CCACCGCTGC CCACGTTGAA 420

CTGAACGGCT GCAAGGCTGT GAACATCGTC ACCGAGAAGG CCTACGATTC CGACACCTAC 480

GATGACTGGA AGGGTAACTT CGATATCCAG AAGCTGAAGG ACAACATCGC TCAGCACGGC 540

GCCGAAAACG TGGTCGCCAT CGTCTCCACC ATCACCTGCA ACTCCGCAGG CGGTCAGCCA 600

GTGTCTATGG CAAACCTGAA GGAAGTTTAC GAGATCGCTA AGCAGCACAA CATCTTCGTT 660

GTGATGGATT CCGCCCGCTT CTGCGAAAAC GCATACTTCA TCAAGGAGCG CGACCCAAAG 720

TACAAGAACT CCACCATCAA GGAGATCATC CTGGATATGT ACAAGTACGC TGACGCCCTG 780

ACCATGTCCG CAAAGAAGGA CCCACTGCTG AACATCGGCG GTCTGGTTTG CATCAAGAAC 840

GACGAAAAGA TCTTCACCCT CGCTCGTCAG CGTTGCGTGC CAATGGAGGG TTTCGTCACC 900

TACGGCGGTC TGGCAGGTCG TGATATGGCA GCTATGGTGC AGGGCCTGGA AGAGGGCGCA 960

GGTGAAGAGT ACCTGCACTA CCGCATCGGC CAGGTCAAGT ACCTGGGTGA CCGCCTGCGC 1020

GAAGGCGGTA TCTCCATCCA GTACCCAACC GGCGGTCACG CTGTGTTCGT TGATTGCAAG 1080

AAGCTGGTTC CACACATCCC AGGTGACCAG TTCCCAGCAC AGGCTGTGAT CAACGCCCTG 1140

TACCTGGAAT CCGGCGTTCG CGCAGTGGAG ATCGGCTCCT TCCTGCTGGG TCGCGATCCA 1200

GAAACCGGCA AGCAGAAGCA CGCTGACATG GAGTTCATGC GCCTGACCAT CGCCCGTCGC 1260

GTTTACACCA ACGATCACAT GGACTACATC GCCGATGCAC TGATCGGTCT GAAGGACAAG 1320

TTCGCTACCC TGAAGGGCCT GGACTTCGAA TACGAGCCAC CAGTTCTCCG TCACTTCACC 1380

GCACGTCTGA AGCCAATCAA GTAA 1404

WP_040595636.1_*Prevotella-pallens_*PpTNA_CO

ATGGAGATCC CATTCGCAGA ATCCTGGAAG ATCAAGATGA TCGAGTCCAT CAAGAAGTCC 60

ACCCGCGAAG AGCGCGAGCA GTGGCTGAAG GAAGCACACT ACAACGTGTT CCAGCTGAAG 120

GCTGAACAGG TCTACATCGA TCTGCTGACC GACTCCGGCA CCGGTGCTAT GTCCGATAAG 180

CAGTGGGCAG CTATGATGCT GGGCGACGAG TCCTACGCCG GTGCAACCTC TTTCTACAAG 240

TTCTCCGAAA CCGTCCAGCG CCTGCTGGGT ATGAAGTACG TTATCCCAAC CCACCAGGGT 300

CGTGCAGCAG AGAACGTGCT GTTCTCCCAC CTGGTGAAGG CTGGCAACGT CATCCCTGGT 360

AACGCCCACT TCGATACCAC CAAGGGCCAC ATCGAGTCCC GCAAGGCTCA CGCCATCGAT 420

GTCACCACCG ATGACGCAAA GGACACCCAG AAGGAAGTCC CATTCAAGGG TAACGTTTGC 480

CTGAACAAGC TGGAGAAGGT TCTGAAGGAA AACAAGGGCA ACGTGCCATT CATGGTTCTG 540

ACCGTGACCA ACAACACCGT TGGCGGTCAG CCAGTGTCCA TGAAGAACAT CAAGGAAACC 600

TGCGCTCTGT GCCACAAGTA CGGTGTCCCA GTTATCATGG ATTCCGCCCG CTTCGCAGAG 660

AACGCTTACT TCATCAAGGT TCGCGAAGAG GGCTACGCCG ACAAGACCAT CAAGGAAATC 720

GTGCAGGAGA TGTACGAAGC TGCCGATGCA GCTACCATGT CCGCAAAGAA GGACGGTGTG 780

GTCAACATGG GCGGTTTCAT CGCCACCAAC AACAAGGAGT GGTTCGAAGG CGCAAAGATG 840

TTCTGCATCC CAATGGAGGG CTACGTGACC TACGGCGGTA TGTCCGGTCG CGATCTGAAC 900

GCACTGGCTC AGGGCCTGGA AGAGAACACC GAATTCGACA TGCTGCAGAC CCGCATCCAC 960

CAGGTCGAGT ACCTGGCTAA GAAGCTGGAT GAATACGGCA TCCCATACCA GCGCCCAGCC 1020

GGCGGTCACG CAATCTTCGT TGACGCCTCC AAGGTCCTGA CCCACGTTCC AAAGGAAGAG 1080

TTCCCAGCTC AGACCCTGAC CTGCGAGCTG TACCTGGAAG CTGGCATCCG CGGCGTGGAA 1140

GTGGGTTACA TGCTGGCAGA TCGCGACCCA GAAACCGGCG AAAACCGCTT CGGCGGTCTG 1200

GATCTGCTGC GCCTGGCTAT CCCACGTCGC GTTTACACCG ACAACCACAT GAACGTTGTG 1260

GCCGCAGCTC TGAAGAACGT GTTCGACCGT CGCGAATCCA TCACCCGCGG CGTCGAAATC 1320

GAGTGGGAAG CACCACTGAT GCGCCACTTC ACCGTCCAGC TGAAGCGCCT GCAGTAA 1377

WP_020497666.1_*Sciscionella-marina_*SmTNA_CO

ATGGAACCAT TCCGCATCAA GTCCGTCGAG CCAATCCCAT TCCCAACCGC TGAAGAACGT 60

CGCAACTCCC TGGCACAGGC AGGTTTCAAC CTGTTCCGCG TTCCAGCACG CCAGGTCACC 120

GTTGATCTGC TGACCGACTC CGGCACCGCA GCTATGTCCG CAGCACAGTG GTCCGCTGTG 180

TTCTCCGGTG ATGAATCCTA CGCTGGCGCC CGCTCCTACG AAGGTTTCGA GGCAGTGGTC 240

CGCGAACTGA CCGGCATGCC AGAGGTCATC CCAGTTCACC AGGGTCGCGC TGCCGAACGC 300

ATCCTGTTCG GCACCCTGCT GCGCCCAGGC GAGATCTCCG TCGCAAACAC CCACTTCGAC 360

ACCACCGCAG CTTCCGTTGC CGCAGCTGGT GCTCGTTGCA TCGATCTGCC AGGCCGCCCA 420

GACCTGCCAG GCAACACCGA AGATTCCGGC CTGTTCGGCG GTAACATCGA CCTGGAAGGT 480

CTGGAACGCG TGCTGCGTGG TCCAGAGAAG GTCCGCTGCG TTGTGCTGAC CGTTACCGAT 540

AACGCTGGCG GTGGCCAGCC AGTCTCCCCA GCTAACCTCG CCGAGGTTCG CCGCCTGTGC 600

GCTCCACGCG GTATCGGTGT GCTGCTGGAT GCATCCCGTT TCGCAGAAAA CGCTTACCTG 660

GTTACCCGCC GCGATCCAGA GTGGGTTCGC GCATCCATCC CAGATGTTGC ACGTGCTATG 720

TTCGATCTGG CCGACGGTTG CTTCGCATCC CTGAAGAAGG ATGGCCTGGC CAACACCGGT 780

GGCCTGATCG CACTGCGCGA TGCTGCACTG GCTCGTGACT GCCGTAACCG TCTGATCGAA 840

GTTGAGGGTT TCCCAACCTA CGGTGGCCTG GCTGGTCGCG ATCTGGAAGC ACTGACCAAG 900

GGTCTGCACG AGGTGATGGA CCCACGCTAC CTGGAATACC GCGCCGAGTC CGCATCCTGG 960

TTCGCAGATC AGCTGGAAAC CGCTGGTTTC CCAGTTCTGC GCCCAACCGG TTGCCACGCT 1020

GTGTACGTCG ACGCTGCCGC AAAGCTGGCA CACATCCCAG CTCCAGAGCT GCCAGCCACC 1080

GCACTGGCTA ACGCCCTGTA CCTGGCAGGT GCTGTTCGCG TGACCGATCT GGGCACCCTG 1140

GTGTTCGGTG GCCCAGATCC AGAAGGTGGC CCAGACCGTC CAGCCCCACG CGAGTGGGTG 1200

CGTTTCGCAC TGCCACGTCG CGTCTACACC CGCAACCACC TGGAATACGT TGCTGACATC 1260

GCTGCACAGG TGGCAGCTAA GGCAACCGCA CTGTCCGGCT ACCGCATCCT GGAACAGGCA 1320

CGTACCCTGC GTCACTTCAC CGCTGTTCTG GCCCCACTGG GCGAACCAGT GCCATTCGAG 1380

CACTAA 1386

WP_011755084.1*_Nocardioides-sp._*NsTNA_CO

ATGGACCAGC CATTCCGCAC CATCATCGAA CCATTCCGCA TCCACTCCGT GGAGCCAATG 60

CGCATGACCA CCGCTGAAGA GCGTCGCACC CACCTGGCAG CTGTCGATTA CAACCTGTTC 120

CAGCTGCGCG CTGAACACGT TCTGATCGAC CTGCTGACCG ATTCCGGCAC CGGTGCAATG 180

TCCCGCGACC AGTGGGCAGC AGTTCAGCGC GGCGATGAGT CCTACGCTGG TTCCCCATCC 240

TACTTCGTTT TCCGCGACGC CGTGCGCCGC CTGTTCGATT TCGAACACAT CATCCCAGTG 300

CACCAGGGTC GTGCTGCTGA GCGCATCCTG TTCTCCGTCC TGGGCGGTGC TGGCAAGGTT 360

ATCCCAAACA ACACCCACTT CGACACCACC CGCGCTAACA TCGAAGCCAC CGGTGCAGAG 420

GCTGTCGATC TGGTTATCGC AGAAGGCCGC GACCCACGCT CCGATCACCC ATTCAAGGGT 480

AACATGGACC TGGCAGCTCT GGAACAGCTG CTGGAGGCCC ACGCAGATGA CGTCCCATGC 540

GTCATGGTTA CCATCACCAA CAACTCCGGC GGTGGCCAGC CAGTTTCCCT CGCAAACCTG 600

CGCGGCGTGC GTGCTCTGTG CGACCGCTTC GGCAAGCCAC TGTTCCTGGA TGCATGCCGC 660

TTCGCAGAAA ACGCTTGGTT CATCCGTGAA CGTGAGGCTG GTCAGGGTGA ACGCGACGTG 720

GTCGATATCA TCCGTGATGT GACCGGTCTG GCTGATGGTG TCACCATGTC CGCAAAGAAG 780

GACCCACTGG GCAACATCGG TGGCTGGCTG GCTCTGGCCG ATGACGATCT GGCCGCACAG 840

TGCCGCAACA TCGTTATCCT GACCGAAGGC TTCCCAACCT ACGGTGGCCT GGCAGGTCGC 900

GACCTGGAAG CACTGGCTCA GGGCCTGGCT GAGGTTGTGC AGCACGATTA CCTGCGCTAC 960

CGCATCGGCT CCACCGCATA CCTGGGTCGT GCACTGGCAG AACGCGGCGT GCCAGTCCTG 1020

TCCCCATTCG GTGGCCACGC AATCTACCTG GATGCTCGTG CACTGCTGCC ACACCTGGAC 1080

CCACTGGAGT ACCCAGGCCA GGCAGTTGCT GTGGCCCTGT ACGAAATCGG TGGCATCCGC 1140

TCCTGCGAGA TCGGCACCGT GATGTTCGGC CGCCACCCAG ATGGTTCCGA GCAGCCAGCT 1200

GCAATGGATC TGGTCCGCCT GGCTATCCCA CGTCGCACCT ACACCCAGTC CCACATCGAC 1260

TACGTGATCG AAGTTTGCGA ACGCATCCTG GACCGTGCAT CCGATCTGCC AGGCTACCGC 1320

ATCGTCGAAG AGCCACCAGC ACTGCGTCAC TTCACCGCAC GTTTCGCACC ACTGGGTCGT 1380

ACCGCATAA 1389

WP_011910553.1_*Rhodobacter-sphaeroides*_RsTNA_CO

ATGACCGTCA AGTTCTTCGG CGGTGACATG GTCCCACTGG AGATGCACAA GGTTCGCGTG 60

GTCCAGAAGC TGACCCTCGC ACCAATCGAA GAACGCCTGC GCGCTATCGC TGAAGCTGGT 120

AACAACACCT TCCTGCTGCA GAACCGCGAT GTTTTCATGG ACATGCTGAC CGATTCCGGT 180

GTGAACGCTA TGTCCGACCG CCAGATGGCA GCTATGATGG TTGCCGATGA CTCCTACGCA 240

GGCTCCTCCA CCTACGAACG CTTCGAGGCT CGCCTGCGCG ACCTGTTCGG CATGGAATGG 300

ATCTTGCCAA CCCACCAGGG TCGCGCCTGC GAGAACATCC TGTCCCAGGT TCTGGTGAAG 360

CCAGGCACCA TCGTCCCAAT GAACTACCAC TTCACCACCA CCAAGGCACA CATCGTTCTG 420

AACGGCGGTT CCATCGAAGA GATCTGCCAC GACCGCGGCC TGGAAGTTAC CTCTACCCAC 480

CCATTCAAGG GTGATATGGA CGTGGGCAAG CTCGAAGGTC TGGTCGCCAC CCACGGCGCA 540

GATCGTATCG CTTTCGTGCG CATGGAAGCA GGCACCAACC TGATCGGCGG TCAGCCAATC 600

TCTATGGCAA ACCTGCGCGA GATCCGTGCT GTGTGCGACG CACACGGCCT GATCTTCGTC 660

CTGGACGCTT CCCTGCTGGC CGATAACCTG TGGTTCATGA AAACCCGCGA AGAGTCCTGC 720

CGCGATCTGT CCATCCCAGA AATCACCCGC CAGGTGGCTG ATCTGTGCGA CGTCATCTAC 780

TTCTCCGCTC GTAAGCTCGG TTGCGCACGC GGCGGTGCTA TCTGCATCCG CTCCGAAGAT 840

CTGTACCGCA AGATGCGCGT CCTGGTTCCA CTGTACGAGG GTTTCCTGAC CTACGGCGGT 900

ATGTCCGTCC GCGAAATCGA GGCACTGACC GTTGGCCTGG AAGAAACTAT GGATACCGAC 960

ATGATCTCCC AGGGTCCACT GTTCATCGAA TACATGGTCA ACGAGCTGGT TAAGCGCGGC 1020

GTGCCAGTCA TCACCCCAGC AGGCGGTCTG GGTTGCCACA TCGATGTTAT GCGCTTCCTG 1080

CCACACGTGC CACAGTCCCA GTACCCAGCT GGTGCACTGG CATCCGCTCT GTACATCGCT 1140

TCCGGCATCC GCGGTATGGA GCGCGGCTCC CTGTCCGAGC AGCGTAACCC AGACGGCACC 1200

GAAGTGTACT CCAACATGGA GCTGCTGCGT CTGGCAATGC CACGTCGCGT TTTCACCCTG 1260

TCCCAGGTGA AGTACGCAAT CGATCGCCTG GACTGGCTGT ACCGCAACCG CGATCTGGTG 1320

GGCGGTCTGG TTTTCGTGGA AGAGCCAGAG ATCCTGCGCT TCTTCTACGG CCGCCTGGAC 1380

CCAGTCGGTG ATTGGCAGCA GAAGCTGGCT GCACGTTTCC GTGCTGATTT CGGCGACTCC 1440

TAA 1443

WP_011220466.1_*Photobacterium-sp._*PsTNA_CO

ATGAAGCGCA TCCCAGAGCC ATTCCGCATC AAGATGGTCG AACCAATCCG CATGACCACC 60

CTGGCAGACC GCGAAGAGGC CCTGAAGCTG GCAGGCTACA ACCCATTCGC TCTGAAGTCC 120

GAGGATGTTT ACATCGACCT GCTGACCGAT TCCGGCACCG GTGCAATGTC CGAGTCCCAG 180

TGGGCTGGCC TGATGCTGGG TGACGAAGCT TACGCCGGCT CCCGCAACTA CTACCACCTC 240

AAGACCGCCG TGGAAGAAGT GTTCGGTTAC CGCGATTTCG TTCCAACCCA CCAGGGTCGC 300

GGTGCTGAGC AGCTGCTGTT CCCATGCCTG GTTGAGAAGA TGCAGCGCGA ACGCGGCGGT 360

AACCACCCAG TGTTCATCTC CAACTTCCAC TTCGACACCA CCGCAGCTCA CGTGGAATTG 420

TCCGGCGCAC AGGCTATCAA CCTGGTCGAT GAGCGCGCTT TCGACACCGA ATCCGATTTC 480

GGCTGGAAGG GTAACATCGA CCTGCAGAAG CTGGAGGATT GCATCTGCCA CTACGGTGTC 540

GAAAACATCG CCGCAATCAT CATGACCATC ACCTGCAACT CCACCGGCGG TCAGCCAGTG 600

TCTATGGCAA ACCTGAAGGC AGTTTACGCT CTGGGCCGCC AGTACCGCAT CCCAGTGGTC 660

ATGGATTCCG CTCGCTTCTG CGAGAACGCC TGGTTCATCA AGCAGCGCGA AGAGGGTGTT 720

CAGTACAAGT CCATCAAGGA AATCGTGCGC CAGATGTTCC GCTACGCCGA CATGCTGACC 780

CTGTCCGCAA AGAAGGACCC AATGGTGAAC ATCGGCGGTC TGTGCGCTGT CCGTGATGAC 840

CCAGAGCTGT TCCAGGCTGT GCAGTCCCGT TGCGTCCCAC TGGAAGGCTT CGTGACCTAC 900

GGCGGTCTGG CAGGTCGTGA CATGGAAGCA CTGGCACGCG GCCTGTTCGA TGGTCTGGAT 960

GAGGACTTCC TGACCTACCG CATCAAGCAG GTCGAATACC TCGGCTCCCG CCTGCACGAT 1020

GCTGGTGTTC CAGTGCAGTG GCCACCAGGC GGTCACGCAG TGTTCGTTGA TGCTAAGAAG 1080

CTGCTGCCAC ACATCCCACC AGAGCGCTTC CCAGCTCAGG CCCTGTGCAA CGCACTGTAC 1140

CTGGAGGCTG GCGTTCGCGC CGTGGAAATC GGCTCCCTGC TGATCGGTCG CGACCCAGTG 1200

ACCAACAACC AGAAGTCCTC CCCATTCGAA TTCATGCGCC TGACCATCCC ACGTCGCGTC 1260

TACACCAACG ATCACATGGA CTACGTTGCA GATGCTGTCA TCGCCGTTGC TCGTTGCGCT 1320

CGTGCACTGA AGGGCCTGGA ATTCGAGTAC GAACCACCAG TTCTGCGCCA CTTCATGGCC 1380

GTGATGCGCC CAGCACAGTA A 1401

WP_036934959.1*_Proteus vulgaris_*PvTNA_CO

ATGGCAAAGC GCATCGTGGA ACCATTCCGC ATCAAGATGG TGGAGAAGAT CCGCGTCCCA 60

TCCCGTGAAG AGCGTGAAGC AGCTCTGAAG GAAGCAGGCT ACAACCCATT CCTGCTGCCA 120

TCCTCCGCTG TCTACATCGA TCTGCTGACC GACTCCGGCA CCAACGCAAT GTCCGATCAC 180

CAGTGGGCCG CAATGATCAC CGGCGACGAA GCCTACGCAG GTTCCCGCAA CTACTACGAT 240

CTGAAGGACA AGGCTAAGGA GCTGTTCAAC TACGATTACA TCATCCCAGC ACACCAGGGT 300

CGCGGTGCAG AAAACATCCT GTTCCCAGTC CTGCTGAAGT ACAAGCAGAA GGAAGGCAAG 360

GCTAAGAACC CAGTTTTCAT CTCCAACTTC CACTTCGATA CCACCGCTGC CCACGTGGAA 420

CTGAACGGTT GCAAGGCAAT CAACATCGTC ACCGAAAAGG CTTTCGACTC CGAAACCTAC 480

GATGACTGGA AGGGCGATTT CGACATCAAG AAGCTGAAGG AGAACATCGC TCAGCACGGT 540

GCCGACAACA TCGTCGCTAT CGTTTCCACC GTGACCTGCA ACTCCGCCGG CGGTCAGCCA 600

GTCTCCATGT CCAACCTGAA GGAAGTTTAC GAGATCGCTA AGCAGCACGG CATCTTCGTG 660

GTCATGGATT CCGCCCGCTT CTGCGAAAAC GCATACTTCA TCAAGGCTCG CGACCCAAAG 720

TACAAGAACG CTACCATCAA GGAAGTGATC TTCGATATGT ACAAGTACGC TGACGCCCTG 780

ACCATGTCCG CAAAGAAGGA CCCACTGCTG AACATCGGCG GTCTGGTGGC CATCCGCGAC 840

AACGAAGAGA TCTTCACCCT GGCACGCCAG CGCTGCGTTC CAATGGAAGG CTTCGTGACC 900

TACGGCGGTC TGGCAGGTCG CGATATGGCA GCTATGGTGC AGGGCCTGGA AGAAGGCACC 960

GAAGAGGAAT ACCTGCACTA CCGCATCGGC CAGGTCAAGT ACCTGGGTGA CCGCCTGCGC 1020

GAGGCAGGCA TCCCAATCCA GTACCCAACC GGCGGTCACG CTGTTTTCGT GGATTGCAAG 1080

AAGCTGGTTC CACAGATCCC AGGCGACCAG TTCCCAGCAC AGGCTGTGAT CAACGCCCTG 1140

TACCTGGAAT CCGGTGTCCG CGCAGTTGAG ATCGGCTCCT TCCTGCTGGG TCGCGATCCA 1200

GCTACCGGCG AACAGAAGCA CGCCGACATG GAGTTCATGC GCCTGACCAT CGCTCGTCGC 1260

GTCTACACCA ACGATCACAT GGACTACATC GCCGATGCAC TGATCGGCCT GAAGGAAAAG 1320

TTCGCCACCC TGAAGGGTCT GGAGTTCGAA TACGAGCCAC CAGTTCTGCG CCACTTCACC 1380

GCACGCCTGA AGCCAATCGA GTAA 1404
